# Caloric restriction attenuates C57BL/6 J mouse lung injury and extra-pulmonary toxicity induced by real ambient particulate matter exposure

**DOI:** 10.1186/s12989-020-00354-2

**Published:** 2020-06-05

**Authors:** Daochuan Li, Shen Chen, Qiong Li, Liping Chen, Haiyan Zhang, Huiyao Li, Dianke Yu, Rong Zhang, Yujie Niu, Shaoyou Lu, Lizhu Ye, Xiaowen Zeng, Guanghui Dong, Rui Chen, Michael Aschner, Yuxin Zheng, Wen Chen

**Affiliations:** 1 Department of Toxicology, School of Public Health, Sun Yat-sen University, 74 Zhongshan Road 2, Guangzhou, 510080 China; 2 Department of Toxicology, School of Public Health, Qingdao University, Qingdao, 266021 China; 3 Department of Toxicology, School of Public Health, Hebei Medical University, Shijiazhuang, 050017 China; 4 Department of Toxicology and Sanitary Chemistry, School of Public Health, Capital Medical University, Beijing, 100069 China; 5 Department of Molecular Pharmacology, Albert Einstein College of Medicine, Forchheimer 209, 1300 Morris Park Avenue, Bronx, NY 10461 USA

**Keywords:** Caloric restriction, Particulate matter, Pulmonary injury, Extra-pulmonary toxicity, RNA sequencing, Xenobiotic metabolism

## Abstract

**Background:**

Caloric restriction (CR) is known to improve health and extend lifespan in human beings. The effects of CR on adverse health outcomes in response to particulate matter (PM) exposure and the underlying mechanisms have yet to be defined.

**Results:**

Male C57BL/6 J mice were fed with a CR diet or ad libitum (AL) and exposed to PM for 4 weeks in a real-ambient PM exposure system located at Shijiazhuang, China, with a daily mean concentration (95.77 μg/m^3^) of PM_2.5_. Compared to AL-fed mice, CR-fed mice showed attenuated PM-induced pulmonary injury and extra-pulmonary toxicity characterized by reduction in oxidative stress, DNA damage and inflammation. RNA sequence analysis revealed that several pulmonary pathways that were involved in production of reactive oxygen species (ROS), cytokine production, and inflammatory cell activation were inactivated, while those mediating antioxidant generation and DNA repair were activated in CR-fed mice upon PM exposure. In addition, transcriptome analysis of murine livers revealed that CR led to induction of xenobiotic metabolism and detoxification pathways, corroborated by increased levels of urinary metabolites of polycyclic aromatic hydrocarbons (PAHs) and decreased cytotoxicity measured in an ex vivo assay.

**Conclusion:**

These novel results demonstrate, for the first time, that CR in mice confers resistance against pulmonary injuries and extra-pulmonary toxicity induced by PM exposure. CR led to activation of xenobiotic metabolism and enhanced detoxification of PM-bound chemicals. These findings provide evidence that dietary intervention may afford therapeutic means to reduce the health risk associated with PM exposure.

## Introduction

Ambient particulate matter (PM) pollution is among the leading four risk factors contributing to deaths and disability-adjusted life-years (DALYs) in China [1]. Emerging evidence has highlighted the association between PM exposure and respiratory diseases, systemic injuries and multiple extra-pulmonary disorders, such as cardiovascular diseases, neurodegenerative diseases, kidney damage, diabetes, colon epithelial injury, etc. [2–8] Although PM levels have been progressively declining in most regions of China, the adverse health effects attributable to PM exposure will continue to rise over at least the next two decades [9]. Thus, intervention modalities are urgently required against the development of diseases associated with PM exposure.

Both epidemiologic and clinical investigations have established strong evidence that dietary modifications, nutrition, and lifestyle have an impact on the onset of diseases associated with environmental toxic insults [10–12]. Healthy dietary and lifestyles have been shown to lower the intensity of environmental stressors associated with adverse health effects by increasing antioxidant and anti-inflammatory mediators [13, 14]. In contrast, individuals with poor dietary habits, such as high intake of processed foods rich in fat and nutrient imbalance, are more susceptible to hazardous chemical-induced health effects [15]. For example, high fat diet or high caloric intake can alter the biological and metabolic activity of individuals, leading to oxidative damage, inflammation and insulin resistance, all of which increase the health risk associated with environmental exposures [16, 17]. Herein, we have posited that modifying dietary habits or nutrient intake would represent a novel approach to protect human beings from health impairment associated with air pollution.

Caloric restriction (CR) is defined as a dietary regimen by reduction of total calorie intake without deprivation of essential nutrients. It has been demonstrated that maintenance of CR is beneficial for health, extending lifespan, reducing weight and the maintenance of metabolic activation [18]. CR also reduces the incidence of cardiovascular diseases, cancers, immune deficiencies, neurodegeneration and diabetes in humans [19–21]. CR affords health benefits by triggering a series of molecular events, including reduction of oxidative damage, acceleration of autophagy, inhibition of inflammation and decreased DNA damage [18, 22]. CR reduces oxidative stress by decreasing the production of reactive oxygen species (ROS) and enhancing the capacity of antioxidants, such as increased activity of antioxidant response elements (ARE) and the levels of glutathione (GSH) [23]. CR has been shown to decrease the levels of circulating pro-inflammatory cytokines, enhance immune functions, and attenuate DNA damage by activation of key components of the DNA-repair machinery, such as recruitment of base excision repair factors, activation of p53 signaling, etc. [22, 24–26]. Moreover, CR could promote anti-tumor effects and inhibit metastases through the induction of autophagy and the modulation of the immune microenvironment [27–29]. Although CR may impact the reduction in PM-induced pulmonary injury and extra-pulmonary toxicity, the underlying mechanisms and the key pathways involved in regulation of cellular functions and integrity has yet to be elucidated.

Previous studies have reported that CR could alter the expression and the activity of a wide range of xenobiotic metabolizing enzymes and transporters. The enhancement of CR-related detoxication of chemical carcinogens, such as aflatoxin B_1_ (AFB_1_), benzo [a] pyrene (BaP), and 7,12-dimethyl-benz(a) anthracene (DMBA), has been linked to the up-regulation of phase-I and phase-II metabolizing genes [30–33]. However, it remains unclear whether the synergistic effects are associated with metabolic activation (primarily phase I enzymes) and detoxification (primarily phase II enzymes), accelerating the clearance of PM-bound chemicals. Moreover, the relationship between the biological effects and specific metabolic pathways has yet to be addressed.

Previously, we established a real-world ambient PM exposure system and showed that high level of PM exposure causes multiple-organ damage in mice [8]. The system we constructed offers optimized conditions to minimize distress and discomfort during the inhalation exposure and replicates a real-ambient PM exposure scenario for experimental animals, resembling the natural state of human exposure to the extent possible. In this study, we examined the effect of CR diet on pulmonary injury and extra-pulmonary effects upon real-ambient PM exposure and explored the underlying molecular mechanisms of CR-mediated biological effects.

## Results

### Characteristics of CR-diet fed mice

**Fig. 1:**
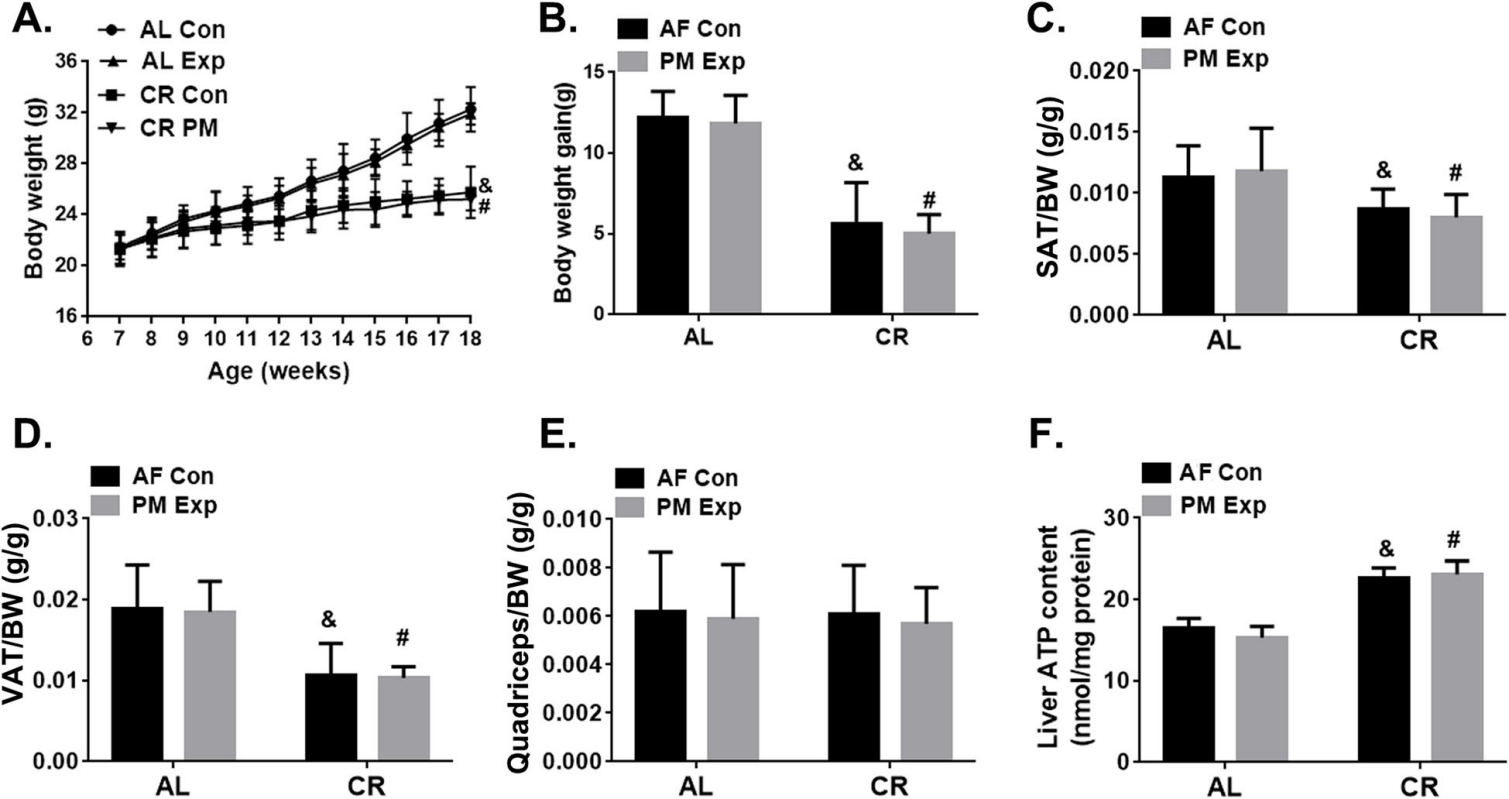
Characteristics of mice fed with CR diet. **a** The dynamic changes in body weight (BW) of AL- and CR-fed mice with (Exp) or without (Con) PM exposure (*n* = 20 per group). **b** The average body weight gain at the end of experiments. Subcutaneous adipose tissue (SAT) weight (**c**), visceral adipose tissue (VAT) weight (**d**), quadriceps weight (**e**) in relative to BW. *n* = 10 per group. **f** Liver ATP content. *n* = 10 per group. The data are expressed as the mean ± SD. ^&^*P* < 0.05 CR-FA vs. AL-FA, ^#^*P* < 0.05 CR- PM vs. AL- PM

In order to adapt to a state of calorie restriction (CR), we gave mice a specific diet to progressively achieve a 40% reduction of calorie intake as described in Method section (**Fig. S****1**). The food intake and water consumption were monitored daily (**Fig. S****2****A-S****2****B**). Compared with ad libitum (AL)-fed mice, CR led to a reduction in body weight (BW) by 20.22 ~ 21.05% (*P* < 0.05) (Fig. 1a-b), in subcutaneous adipose tissue (SAT) by 23.30 ~ 32.20% (*P* < 0.05) (Fig. 1c), and in visceral adipose tissue (VAT) by 43.39 ~ 44.32% (*P* < 0.05) (Fig. 1d). Notably, CR conferred mice with absent signs of malnutrition or lean mass loss (Fig. 1e). CR also increased the levels of ATP in mouse liver by 18.96 ~ 25.24% (*P* < 0.05), indicating enhanced mitochondrial function (Fig. 1f). As indicated by the biochemical analysis of plasma (**Fig. S****3****A, B, D, E**), the levels of cholesterol (CHOL), triglyceride (TG), low-density lipoproteins cholesterol (LDL-C), and glucose (GLU) in CR mice were significantly reduced by 19.55 ~ 24.81%, 22.99 ~ 26.32%, 23.81 ~ 25.00%, 14.59 ~ 18.99%, respectively. In addition, the levels of glutamic-pyruvic transaminase (ALT), glutamic-oxalacetic transaminase (AST) in CR-fed mice has no significant difference compared to those in AL-fed mice (*P* > 0.05) (**Fig. S****3****F-G**). The successful generation of the CR mouse model provided us a valuable tool to further investigate the effects of CR on PM-induced toxicity.

### Characterization of real ambient PM exposure

Whole-body PM inhalation was conducted in a real-ambient PM exposure system for 4 weeks from Jan 4th to Feb 1st, 2018 in Shijiazhuang, China (**Fig. S****4**). Daily PM_2.5_ concentration of ambient air and exposure chamber were monitored (**Fig. S****5**). The daily mean concentration of PM_2.5_ in ambient air was 151.07 μg/m^3^, which was 4.32-fold higher than the daily average limit of 35 μg/m^3^ (Air Quality Guidelines of China), and 15.11-fold higher than the daily average limit of 10 μg/m^3^ (Air Quality Guidelines of the World Health Organization) (Table 1**)**. In the course of 4-week PM exposure, the mean concentration of PM_2.5_ in the exposure chamber was 95.77 μg/m^3^, which was 63.40% of the ambient air. The cumulative exposure burden in mice lung was estimated at 31.32 μg /mouse by using the Multiple-Path Particle Dosimetry (MPPD) model (Table 1). As shown in **Fig. S****6**, the size of PM in the exposure chamber ranged mostly from 0.5 to 1.5 μm. In contrast, the PM_2.5_ in the AF control chambers were undetectable.

**Table 1:** The mean PM2.5 concentration and cumulative lung burden during the exposure period

Mean daily PM2.5 concentration (μg/m^3^)		Estimated cumulative lung burden ^a^ (μg/mouse)	35 < PM2.5 ≤ 150 μg/m^3^ (Days)	PM2.5 > 150 μg/m^3^(Days)
Chamber	Ambient			
95.77	151.08	31.32	12	14

**Note**: ^a^Estimated cumulative lung burden = MV × T × CON×DF. *MV* minute ventilation (mL/min); *T* total exposure time (min); *CON* mean concentration (mg/m^3^); *DF* pulmonary deposition fraction (m^3^), DF is estimated by MPPD 3.04

Atmospheric PM_2.5_ was collected daily and quantitative analysis was conducted to characterize the chemical composition of PM_2.5_. To characterize the organic components of PM_2.5_, we determined PM2.5-bound polycyclic aromatic hydrocarbons (PAHs), nitro derivatives of PAHs (nitro-PAHs), alkyl derivatives of PAHs (alkyl-PAHs), polychlorinated dibenzo dioxins (PCDDs), polychlorinated biphenyls (PCBs). As shown in **Table S****4****-S****7**, the sums of PAHs, nitro-PAHs, alkyl-PAHs, PCDDs and PCBs were 154.07 ng/m^3^, 0.759 ng/m^3^, 279.71 ng/m^3^, 0.584 pg/m^3^, 6.101 pg/m^3^, respectively. Specifically, the mean concentration of benzo [a] pyrene (BaP), PCDF, PCDD far exceeded the daily limit of Air Quality Standards of China (**Table S****9**). Moreover, the metal elements and anions were also analyzed, and the sums of metal elements and anions were 3.57 × 10^3^ ng/m^3^, 3.12 × 10^4^ ng/m^3^ (**Table S****8****)**. The levels of chromium (Cr) and arsenic (As) far exceeded the daily limit (**Table S****9**). Taken together, the location of this PM exposure system was representative of the heavily PM-polluted areas in China.

### CR efficiently protected against mouse pulmonary injury induced by PM exposure

**Fig. 2:**
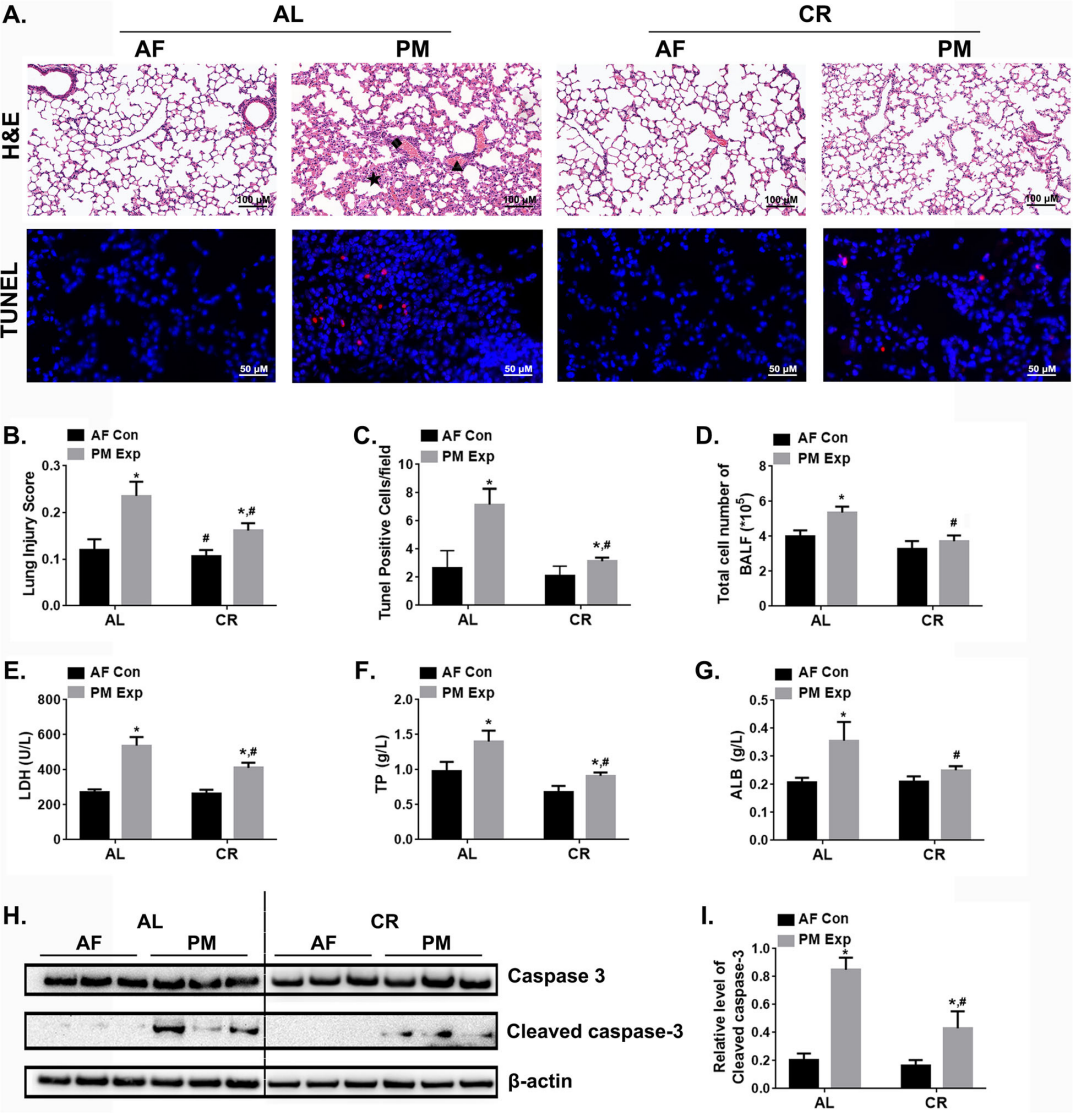
CR protects against PM-induced pulmonary injury. Al-fed and CR-fed mice were exposed to PM for 4 weeks. **a** Representative images of H&E staining (magnification, 200×) and TUNEL staining of lung tissues (magnification, 400×) in different groups of mice. The typical pathological changes, including neutrophil infiltration (★), alveolar septal thickening (◆), alveolar hemorrhage (▲) were indicated. The lung injury scores (**b**), the number of tunnel positive cells (**c**) in mouse lung tissues. n = 10 per group. The total cell number (**d**), the levels of lactate dehydrogenase (LDH) (E), the total protein contents (TP) (**f**), and albumin contents (ALB) (**g**) in mouse bronchoalveolar lavage fluid (BALF). *n* = 10 per group. **h**-**i** Immunoblotting analysis presents the levels of cleaved caspase-3 in mouse lung tissues. *n* = 3 per group. The data are expressed as the mean ± SD. ^*^P < 0.05 (AF vs. PM), ^&^*P* < 0.05 (CR-AF vs. AL-AF), ^#^*P* < 0.05 (CR-PM vs. AL-PM)

To assess the effects of CR on pulmonary injury in response to PM exposure, we conducted histological examination and bronchoalveolar lavage fluid (BALF) analysis in mice. The histopathological examination revealed that PM exposure induced interstitial infiltration of neutrophils, alveolar septal thickening, and alveolar hemorrhage in AL-fed mice, whereas moderate pathologic injury was observed in CR-fed mice (Fig. 2a). As indicated by the pulmonary injury score (Fig. 2b), PM exposure led to a 77% increase of pulmonary injury in AL-fed mice compared to the AF control group, while 45% increase was observed in CR-fed mice. Consistent with the pathological changes, CR remarkably alleviated the pulmonary injury upon PM exposure in terms of total cell number, total protein (TP) content and albumin (ALB) levels, as well as the release of lactate dehydrogenase (LDH) in BALF compared to AL-fed mice (Fig. 2d-g). In addition, PM exposure led to increased number of TUNEL-positive cells (apoptotic) in AL mice by 73.32%, but no significant change in CR-fed mice (Fig. 2a, c). Correspondingly, the level of cleaved caspase-3 was reduced by 49.41% upon PM exposure in CR-fed mice compared to AL-fed mice (Fig. 2h, i). Taken together, these observations indicate that CR significantly alleviates pulmonary injury in response to PM exposure.

### CR alleviated PM-induced pulmonary oxidative stress, genotoxic damage and inflammatory response

**Fig. 3:**
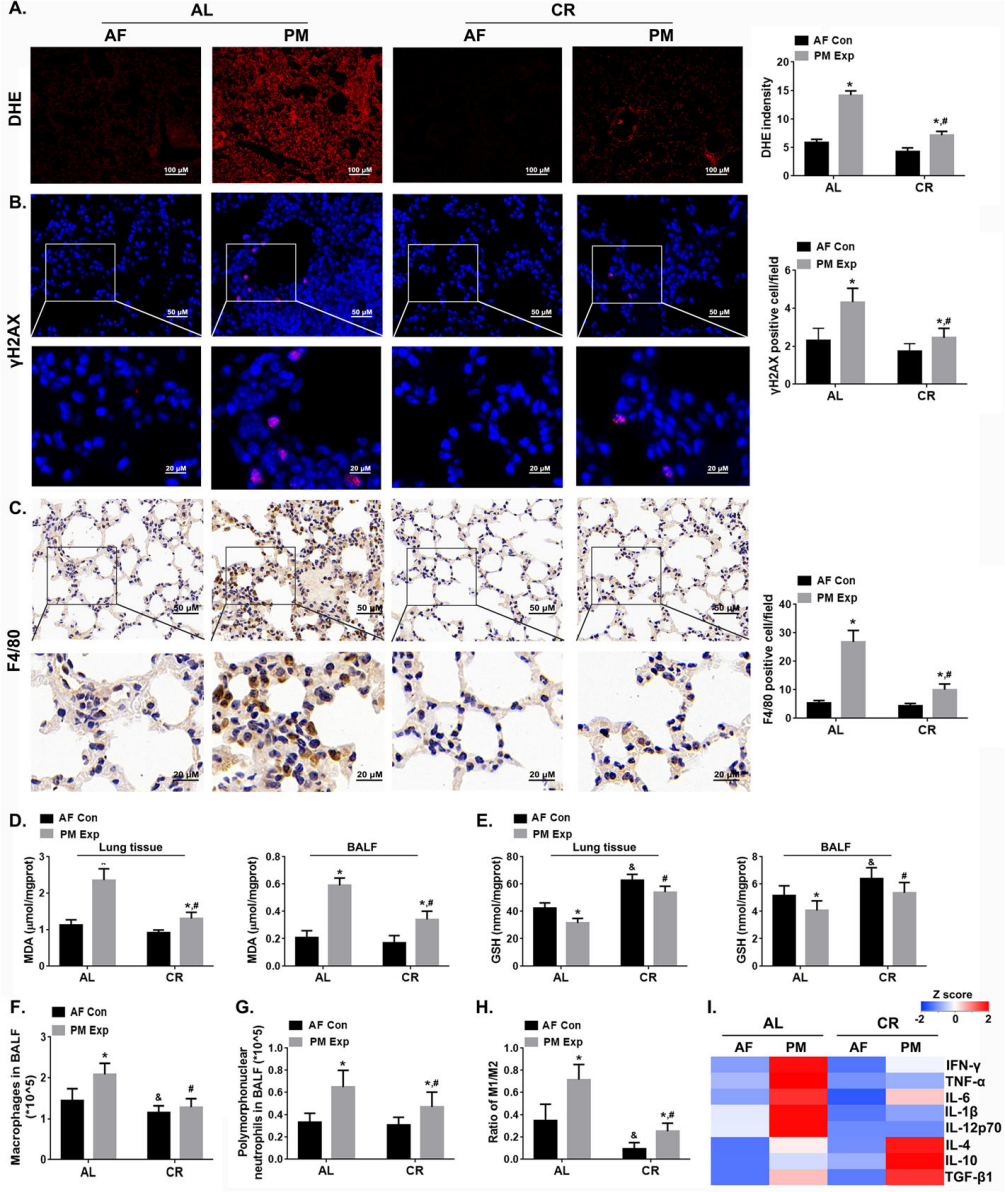
CR led to attenuation of PM-induced oxidative stress, DNA damage and inflammatory response in mouse lung. Representative images and quantitation of DHE (magnification, 200×) (**a**), γH2AX staining (**b**), F4/80 staining (**c**) (magnification, 400× & 1000×). *n* = 10 per group. The contents of MDA (**d**) and GSH (**e**) in mouse lung tissue and BALF, respectively. *n* = 10 per group. The number of macrophages (**f**) and polymorphonuclear neutrophils (PMN) (**g**) in BALF. *n* = 10 per group. **h** The M1 and M2 macrophage polarization in cells isolated from BALF. *n* = 5 per group. The data are expressed as the mean ± SD, ^*^P < 0.05 (AF vs. PM), ^&^*P* < 0.05 (CR-AF vs. AL-AF), ^#^*P* < 0.05 (CR-PM vs. AL-PM). **i** The heatmap illustrates the relative levels of cytokines examined in BALF, including IFN-γ, TNF-α, IL-6, IL1-β, IL-12p70, IL-4, IL-10, and TGF-β1. The detailed information of the cytokine levels was shown in **Fig. S****8**. The z-score transformation was utilized to calculate the relative level of cytokines according the following equation: Z= $\frac{X-\overline{X}}{SD}$ ($\overline{X}$ is the mean value, SD is standard deviation). *n* = 5 per group

Next, we assessed the effects of CR on oxidative stress, genotoxicity and inflammatory response upon PM exposure. Compared to the AL-fed mice, the levels of ROS and malondialdehyde (MDA) were lower in the lung tissues and BALF of CR-fed mice upon PM exposure (Fig. 3a, d). A 21.35 ~ 25.50% decrease in glutathione (GSH) content was observed in AL-fed mice upon PM exposure, but only 13.83 ~ 16.16% decrease was observed in CR-fed mice (Fig. 3e). To determine genotoxicity, we performed immunofluorescence (IF) analysis of γH2AX, a marker of the double strand breakage (DSB). As shown in Fig. 3b**,** γH2AX-postive cells were significantly increased by 87.13% in AL-fed mice after PM exposure, while no significant change was observed in CR-fed mice. Pulmonary pro-inflammatory and anti-inflammatory effects were determined by immunohistochemistry (IHC) staining of F4/80, M1/M2 polarization, and cytokine analysis. The F4/80-positive cells, a marker of macrophage recruitment, were significantly increased by 3.04 folds and 1.27 folds in AL-fed and CR-fed mice upon PM exposure, respectively (Fig. 3c). Similar results were obtained when counting the number of macrophages and polymorphonuclear neutrophils (PMNs) in BALF (Fig. 3f, g). Moreover, we determined the polarization of M1 and M2 macrophages in BALF, which indicated pro-inflammatory and anti-inflammatory effects, respectively. The results showed a significant increase in the proportion of M2 polarized macrophages in CR-fed compared AL-fed mice in response to PM exposure (Fig. 3h, **Fig. S****7****A-C**). Consistent results were observed in the mRNA expression of M1 or M2 macrophage markers in BALF cells (**Fig. S****7****D-E**). Importantly, the secretion of cytokines (IFN-γ, TNF-α, IL-1β, and IL-12p70) in BALF, which is linked to the potency of pro-inflammation and M1 macrophage polarization increased in AL-fed mice (Fig. 3i, **Fig. S****8****A-S8E**). However, the levels of IL-4, IL-10, and TGF-β1, which are indicative of the anti-inflammatory capacity and M2 macrophage polarization, were notably higher in CR-fed mice exposed to PM (Fig. 3i, **Fig. S****8****F-S****8****H**). Taken together, these observations demonstrate that CR leads to reduction in oxidative damage, DNA damage, and pro-inflammation, contributing to the protective effects against PM-induced pulmonary injury.

### The effects of CR on regulation of pathways involved in the protective effects towards PM exposure in mouse lung

**Fig. 4:**
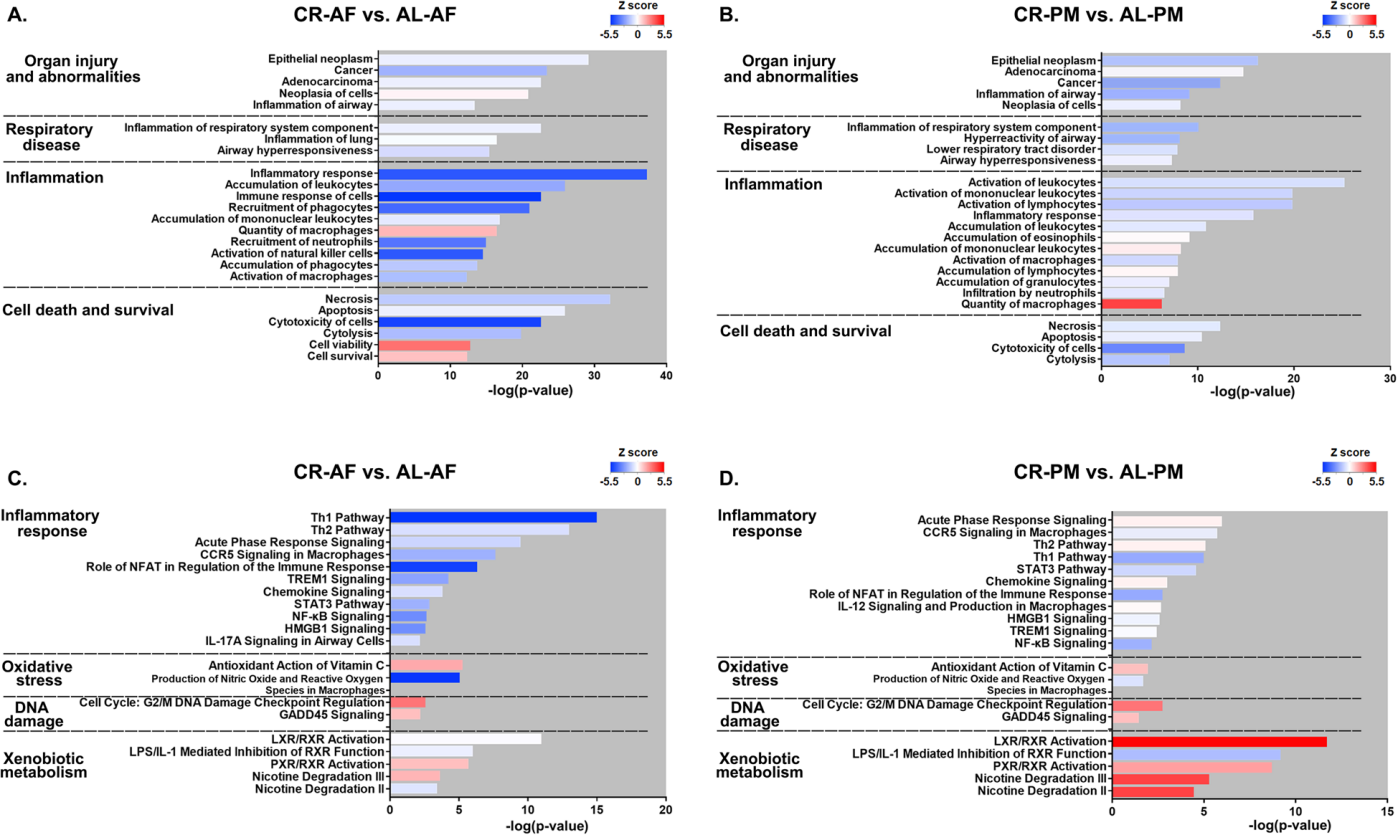
Signature of gene expression and functional alternations altered by CR in mouse lung tissues. Ingenuity Pathway Analysis (IPA) reveals differentially expressed genes (DEGs) in mouse lung tissues of AL- and CR-fed mice with or without PM exposure. The comparison of the categories of disease and toxicological effects involved in organ injury and abnormalities, respiratory disease, inflammation, cell death and survival between CR-AF and AL-AF mice (**a**) and between CR-PM and AL-PM mice (**b**). The comparison of the key canonical pathways involved in inflammatory response, oxidative stress, DNA damage, and xenobiotic metabolism between CR-AF and AL-AF (**c**) and between CR-PM and AL-PM mice (**d**). *n* = 3 per group

Transcriptome profiling of lung tissue was performed to elucidate the molecular mechanisms underlying the effects of CR on PM-induced pulmonary injury. Initial analysis identified 2299 and 1928 differentially expressed genes (DEGs) based on the comparison between CRC and ALC (AF control group) and between CRE and ALE (PM exposed group). Detailed information of the DEGs is shown in **Fig. S****9**. To address the biological relevance of the modifications in the gene signature profiles, we employed IPA software to identify critical biological functions and molecular pathways. As a result, IPA analysis identified approximately 500 biological functions, diseases and toxicological outcomes related to the identified DEG profiles. The most related diseases and biological functions predicted were categorized targeting organ injury and abnormalities, inflammatory response, cell death and survival, cell-to-cell signaling and interaction, and respiratory disease (Fig. 4a, b). Notably, the biological function changed in CR-fed mice, with or without PM exposure, implicating a reduction in inflammation, accumulation, recruitment and activation of immune cells (leukocyte, monocyte, lymphocyte, neutrophils, etc.), and cytotoxicity (necrosis, apoptosis, cytolysis), concomitant with increased cell viability. These signatures in gene expression corresponds to the biochemical and histologic changes that are attributable to CR (Fig. 2-3).

Next, we analyzed canonical pathways associated with the CR-mediated protective effects. We identified 542 and 528 pathways significantly altered between CRC and ALC groups, and between the CRE and ALE groups, respectively. The 20 most significantly altered pathways are shown in **Fig. S****10**. Of the pathways identified, we paid special attention to the those related to inflammatory response, oxidative stress, DNA damage and xenobiotic metabolism (Fig. 4c, d). Notably, we found that CR led to a decline in the activity of several pathways, including acute phase response signaling, Th1 pathway, chemokine signaling, and several key molecular pathways involved in inflammatory regulation in mice with or without PM exposure, such as nuclear factor-kappaB (NF-κB), C-C chemokine receptor type 5 (CCR5), signal transducer and activator of transcription 3 (STAT3), nuclear factor of activated T cells (NFAT), interleukin12 (IL-12), high mobility group protein B1 (HMGB1), triggering receptor expressed on myeloid cells 1 (TREM1), and IL-6 pathway, etc. Specifically, pathways mediating antioxidant functions were activated, and those associated with nitric oxide and reactive oxygen generation were inhibited in CR-fed mice with or without PM exposure. In addition, DNA damage checkpoint regulation and growth arrest and DNA damage gene 45 (GADD45) signaling were activated in CR-fed mice of both the AF control and the PM exposure group, indicating that the capacity of DNA repair was enhanced under the state of CR. The activation of retinoic acid receptors (RXR) might be involved in detoxification of xenobiotics. The major genes in the key canonical pathways of murine lung are shown in **Table S****10** and were selected for validation by qPCR as shown in **Fig. S****11**. Collectively, the modifications in transcriptomic profiles and specific pathway regulations were in agreement with the biological effects of CR, corroborating the notion that CR played an important role in attenuating PM-induced pulmonary injury.

### CR attenuates the extra-pulmonary toxicity upon PM exposure

**Fig. 5:**
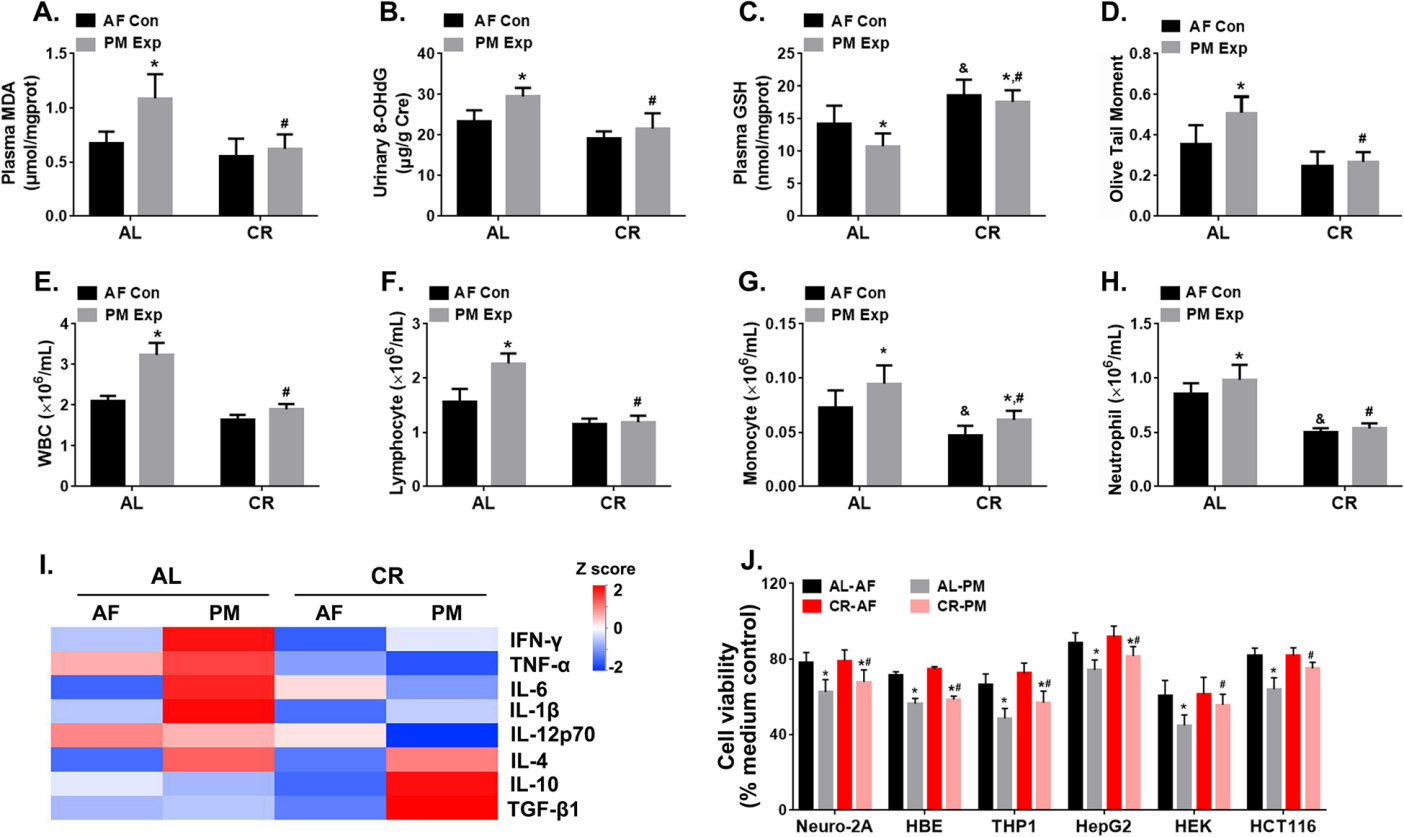
Effects of CR on PM-induced extra-pulmonary toxicity. The contents of MDA in plasma (**a**), 8-OHdG in urine (**b**), and GSH in plasma (**c**) of AL- and CR-fed mice with (Exp) or without (Con) PM exposure. *n* = 10 per group. **d** The olive tail moment of peripheral blood cells (Comet assay). *n* = 10 per group. The cell counts of white blood cells (WBC) (**e**), lymphocytes (**f**), monocytes (**g**), and neutrophils (**h**) in peripheral blood. *n* = 10 per group. **i** The heatmap illustrates the relative levels of cytokines in plasma, including IFN-γ, TNF-α, IL-6, IL1-β, IL-12p70, IL-4, IL-10, and TGF-β1. The detailed information of the cytokine levels was shown in **Fig. S****12**. The z-score transformation was utilized to calculate the relative level of cytokines according the following equation: Z= $\frac{X-\overline{X}}{SD}$ ($\overline{X}$ is the mean value, SD is standard deviation). *n* = 5 per group. **j** Cytotoxicity of mouse plasma on different human cell lines, including Neuro-2A, HBE, THP1, HepG2HEK, and HCT116 were determined by ex vivo assay. The cells without an addition of the plasma from the mice were regarded as negative control (100% cell viability). *n* = 5 per group. The data are expressed as the mean ± SD, ^*^P < 0.05 (AF vs. PM), ^&^*P* < 0.05 (CR-AF vs. AL-AF), ^#^*P* < 0.05 (CR-PM vs. AL-PM)

To assess extra-pulmonary abnormalities in response to PM exposure, we compared oxidative stress levels, genotoxicity and inflammatory responses between AL- and CR-fed mice with or without PM exposure. The results showed that the levels of plasma MDA and urinary 8-hydroxydeoxyguanosine (8-OHdG) were increased by 61.02 and 26.58% in AL-fed mice following PM exposure, while no significant change was observed in CR-fed mice (Fig. 5a, b**)**. In addition, higher concentration of serum GSH was detected in CR-fed mice, reflecting improved redox status in CR-fed mice (Fig. 5c). To assess the degree of DNA damage, we conducted comet assays in peripheral blood cells and found that the olive tail moments in AL-fed mice were extended by 43% (Fig. 5d). In contrast, there was no difference between the PM exposed and control groups of CR-fed mice. Moreover, the cell numbers of peripheral white blood cell (WBC), lymphocyte, monocyte, and neutrophil were reduced by 54.96, 45.74, 29.68, 15.20%, respectively, in CR-fed mice compared to those in the AL-fed mice following PM exposure (Fig. 5e-h). The anti-inflammatory cytokines (IL-4, IL-10 and TGF-β1) were significantly up-regulated, while the pro-inflammatory cytokines (IFN-γ, TNF-α, IL-1β, IL-6, and IL-12p70) were slightly induced in CR-fed mice compared to AL-fed mice upon PM exposure (Fig. 5i, **Fig. S12**). However, no significant difference appeared in CR-fed mice following PM exposure in terms of the contents of CHOL, TG, LDL-C, and GLU compared to the AL-fed mice (**Fig. S3A, B, D, E**). Notably, the levels of ALT and AST were significantly lower in CR-fed mice compared to the AL-fed mice upon PM exposure (**Fig. S3F-S3G**), indicating that CR might ameliorate the PM-induced liver injury.

Since the in vitro cytotoxicity has been linked to the severity of the pathological injury of multiple organs [8, 34, 35], we conducted ex vivo assays to examine the cytotoxicity of mouse plasma isolated from mice with or without PM exposure in multiple cell lines, including Neuro-2A, HBE, THP1, HepG2, HEK, and HCT116. As shown in Fig. 5j, we found a reduction in cell viability ranging from 16.32 to 27.13% (AL-fed mice) and from 8.36 to 16.28% (CR-fed mice), respectively in multiple cells treated with the diluted plasma following PM exposure, indicating that a decreased toxicity presented in peripheral blood of CR-fed mice. Taken together, these findings demonstrate that CR leads to an abatement in the PM-induced extra-pulmonary toxicity.

### CR enhanced xenobiotic metabolism and detoxification in mouse liver

**Fig. 6:**
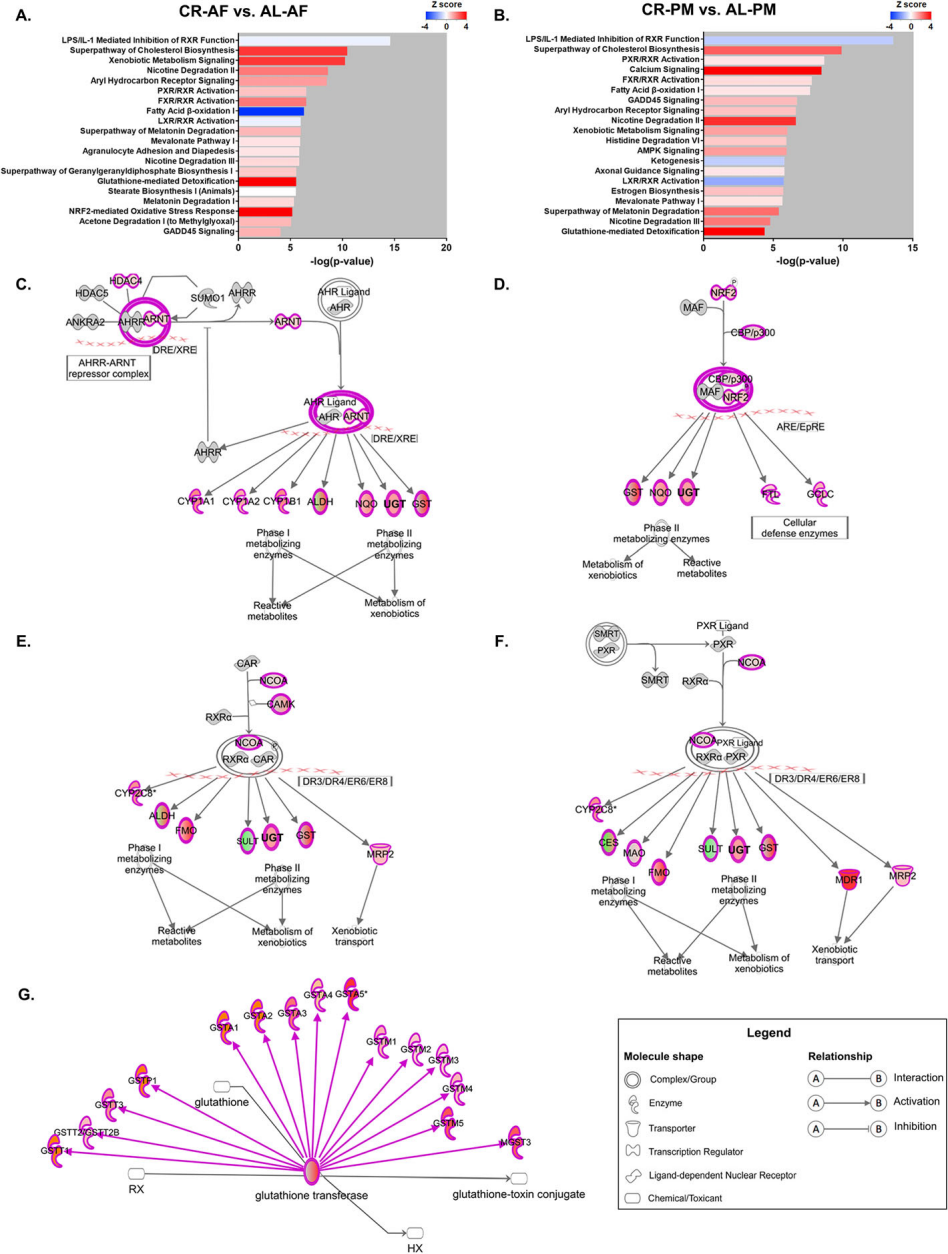
Perturbation of canonical pathways were reversed by CR in mouse livers. Comparison of the 20 most significant canonical pathways identified by IPA analysis between CR-AF and AL-AF mice (**a**) and between CR-PM and AL-PM mice (**b**). Molecular pathways involved in xenobiotic metabolism include AhR signaling (**c**), NRF2-mediated oxidative stress response (**d**), RXR activation (**e-f**), and glutathione-mediated detoxification (**g**). Circles filled in pink colors indicate a trend of upregulations, while circles filled in green colors indicate a trend of downregulations in CR-fed mice with or without PM exposure. *n* = 3 per group

Metabolic activation and detoxification of environmental chemicals in liver play a key role in cellular toxicity and organ injuries [36]. To examine the effects of CR on xenobiotic metabolism, we performed transcriptome profiling analysis in mouse liver tissues. 2821 and 2995 DEGs were identified between CRC and ALC groups and between CRE group and ALE groups, respectively. Detail information on the DEGs is shown in **Fig. S13**. In addition, 611 and 601 canonical pathways were identified by a comparison between AL-AF and CR-AF mice and between AL-PM and CR-PM mice, respectively. Of the 20 most significantly altered pathways, we found that the activation of NRF2-mediated oxidative stress response and GADD45 signaling were specifically inherent to the CR-fed mice (Fig. 6a-b), suggesting enhanced capacity of antioxidant and more efficient DNA-repair contribute to the attenuation of PM-induced systemic toxicity. Notably, the activity of metabolic pathways, including xenobiotic metabolism signaling, aryl hydrocarbon receptor (AhR) signaling, nuclear factor E2-related factor 2 (NRF2)-mediated metabolism, glutathione-mediated detoxification, and RXR activation pathways were significantly enhanced in CR-fed mice with or without PM exposure. The identified pathways involved in xenobiotic metabolism are shown in Fig. 6c-g. Particularly, the activation of AhR signaling is associated with increased levels of phase I metabolizing enzymes including cytochrome P450 1A1 (CYP1A1), CYP1A2, CYP1B1, and aldehyde dehydrogenase (ALDH) and phase II metabolizing enzymes including NAD(P) H quinone dehydrogenase (NQO), UDP-glycosyltransferase (UGT), and glutathione S-transferase (GST) in CR-fed mice (Fig. 6c). The upregulation of NRF2-mediated metabolism and glutathione-mediated detoxification led to higher levels of phase II metabolizing enzymes (NQO, UGT, GST, etc.) in CR-fed mice (Fig. 6d, g). In addition, we found that the activated RXR pathway induced by CR are related to the upregulation of phase I metabolizing enzymes such as CYP2C8, ALDH, and flavin-binding monooxygenase (FMO) and phase II metabolizing enzymes, such as UGT and GST, as well as transporters such as mitochondrial ribosomal protein 2 (MRP2) and multidrug resistance gene 1 (MDR1) (Fig. 6e-f). The major genes in the key canonical pathways of mouse liver are listed in **Table S****11** and the validation of gene expression was performed by qPCR (**Fig. S****14****)**. The changes in enzyme activity induced by CR are critical for determining metabolic activation and detoxification of PM-bound chemicals.

**Fig. 7:**
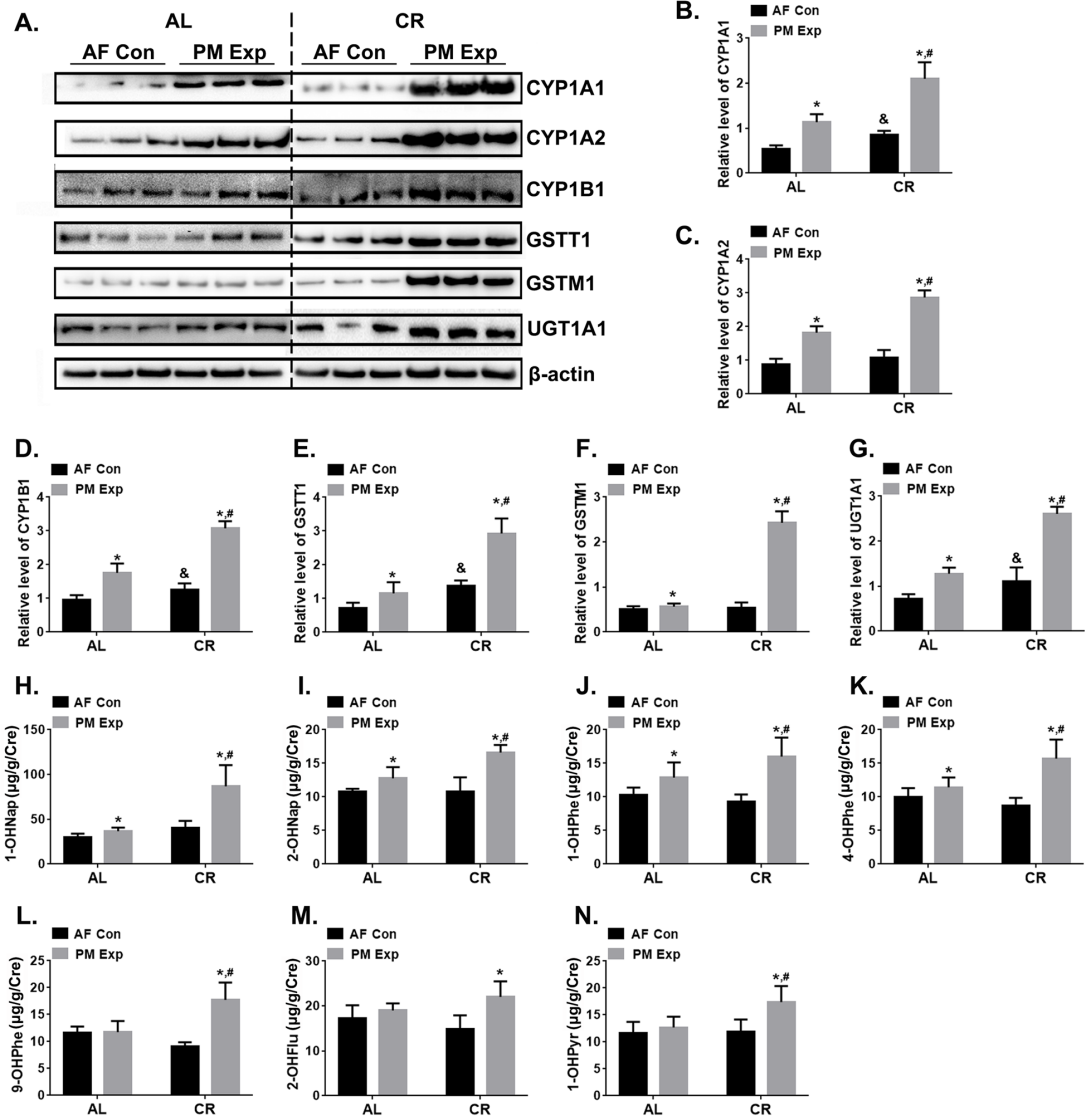
CR enhances the activities of xenobiotic metabolism and detoxification of PM-bound PAHs. **a** The induction of CYP1A1, CYP1A2, CYP1B1, GSTT1, GSTM1, and UGT1A1 in mouse liver tissues of AL- and CR-fed mice with (Exp) or without (Con) PM exposure. *n* = 3 per group. The quantitative analysis of CYP1A1 (**b**), CYP1A2 (**c**), CYP1B1 (**d**), GSTT1 (**e**), GSTM1 (**f**), and UGT1A1(**g**) in different groups of mice. The concentration of urinary hydroxylated metabolites of PAHs (OH-PAHs), 1-OHNap (**h**), 2-OHNap (**i**), 1-OHPhe (**j**), 4-OHPhe (**k**), 9-OHPhe (**l**), 2-OHFlu (**m**), and 1-OHPyr (**n**) in different groups of mice. *n* = 3 per group. The data are expressed as the mean ± SD, ^*^P < 0.05 (AF vs. PM), ^&^*P* < 0.05 (CR-AF vs. AL-AF), ^#^*P* < 0.05 (CR-PM vs. AL-PM)

To further address the effects of CR on metabolic enzyme activity and the correlation with the removal of toxic chemicals, we examined the levels of metabolic enzymes towards polycyclic aromatic hydrocarbons (PAHs), a major category of PM-enriched chemicals. As shown in Fig. 7a-g, immunoblotting analysis revealed that the phase I (CYP1A1, CYP1A2, and CYP1B1) and phase II (GSTT1, GSTM1, and UGT1A1) metabolizing enzymes involved in PAHs metabolism were all upregulated in the liver of CR-fed mice with or without PM exposure, consistent with the results from the transcriptome analysis. The urinary hydroxylated metabolites of PAHs (OH-PAHs) were quantified by GC-MS. We found that the urinary concentration of 1-OHNap, 2-OHNap 1-OHPhe, 4-OHPhe, 9-OHPhe, 1-OHPyr were significantly higher in the PM exposure group of CR-fed mice than AL-fed mice (Fig. 7h-m). Taken together, these findings reveal that CR plays an important role in changes of metabolic activation of PM-bound chemicals. The enhanced expression of phase II metabolizing enzymes triggered by CR might promote a rapid detoxification of toxicants and likely reduced health risks associated with PM exposure.

## Discussion

Dietary, nutritional and lifestyle interventions have been shown to promote health by lowering toxicant burden in both animals and humans [11, 13]. Growing evidence has established that CR represents an effective intervention against a variety of adverse health outcomes [18, 37]. In this study, we demonstrated that CR confers murine resistance against PM-induced pulmonary injury and extra-pulmonary toxicity. The afforded protection is characterized by reduction in oxidative stress, genotoxicity, and inflammatory responses. Transcriptomic analysis further corroborated the efficacy of CR to activate protective molecular pathways. Notably, altered xenobiotic metabolism and detoxification triggered by CR facilitate the metabolic activation and detoxification of PM-bound chemicals such as PAHs. These findings provide novel insight into the mechanism by which CR mediates the protective roles against PM-induced toxicity and suggest that CR represents a powerful approach for intervention against air pollution-related health injury.

Dietary practices are well-defined modulators of human health and biological effects, thereby having a great impact on disease susceptibility in response to environmental stress [13, 38, 39]. Recent findings indicate that poor dietary habits, such as high intake of caloric carbohydrate- or fat-rich diet contribute to metabolic dysfunction and systemic pro-inflammation [16, 40, 41], leading to increased susceptibility to adverse health effects in response to air pollution [42, 43]. In contrast, chronic reduction of caloric intake, in the absence of malnutrition, has been reported to improve lifespan and have anti-cancer effects [18]. In the present study, we demonstrated that CR attenuated the pulmonary injury and extra-pulmonary toxicity associated with murine exposure to PM. These effects are mediated, at least in part, by the enhancement of the antioxidants, DNA damage repair, and anti-inflammatory capacity in response to PM exposure.

Although previous studies have shown that CR modulates the redox status [40], maintains the genome integrity [44–46], reduces inflammatory activity, and improves immune function [24, 47], the molecular mechanisms underlying CR-triggered alterations in a variety of biological functions have yet to be determined. With the aid of transcriptional profiling and analysis of pathway pertubation, we were able to systematically illustrate the molecular regulatory networks that contribute to CR-induced antagonistic effects against PM exposure-induced damage. Upon CR, pathways involved in antioxidant functions could be activated to inhibit the generation of ROS and trigger antioxidant enzymatic activity [23]. In this study, analysis of gene expression profiles revealed that multiple key pathways were activated in CR-fed mice with or without PM exposure, corroborating the reduction in nitric oxide and ROS levels, enhanced activity of the antioxidant vitamin C, Nrf2-mediated oxidative stress response, and glutathione-mediated detoxification, which were corresponding to the reduction of oxidative damage in CR-fed mice.

CR facilitates the maintenance of genome integrity by accelerating DNA repair, thus leading promoting cellular survival and longevity [44–46]. In this study, we demonstrate that a major consequence of ambient PM exposure was associated with pulmonary and extra-pulmonary genotoxicity, which was reversed in CR-fed mice. Previous studies have shown that CR decreased γH2AX foci formation, a marker of DSB in DNA repair deficiency mice, and increased the level and activity of p53 through promoting DNA-dependent protein kinase (DNA-PK) activation, and accelerating cellular DNA repair capacity [26, 48, 49]. Herein, we identified specific pathways, such as GADD45 pathway that was activated by CR and might contribute to the promotion of DNA repair. G2/M DNA damage checkpoint regulator, DNA damage protein 45 (GADD45) signaling is critical in pulmonary and hepatic growth arrest. Activation of GADD45 pathway is involved in stimulation of DNA repair through interaction with cyclin-dependent kinase 1 (Cdk1) [50, 51]. Taken together, CR robustly protects against the PM-induced genotoxicity and promotes genome integrity by improving the DNA repair capacity.

CR is known to inhibit inflammatory responses and to improve immunity [24, 47]. Prior studies have indicated that CR inhibited critical pathways involved in cytokine and chemokine signaling to reduce the level of circulating pro-inflammatory cytokines, such as TNFα, IL-6, and IL-1β [52–57]. Healthy individuals undergoing short-term CR have been shown to have reduced monocyte metabolic and inflammatory activity and a drastic decrease in circulating and organ monocyte numbers [58, 59]. The recruitment of inflammatory cells in tissues and airway induced by intake of high fat or high caloric diet could be reversed by CR [60, 61]. In this study, we establish that CR could reduce the levels of pulmonary and circulating inflammatory triggers. Notably, we verified that the inhibition of inflammatory pathways, such as NF-κB, CCR5, STAT3, NFAT, IL-12, HMGB1, TREM1, and IL-6 might be involved in CR-mediated anti-inflammatory effects upon PM exposure.

Xenobiotic metabolism plays a vital role in mediating the toxicity of PM exposure [62]. Dietary intake could interact with xenobiotic response elements (XREs) to regulate the expression and activity of metabolizing enzymes, thus modifying the cellular response to xenobiotic stressors [63, 64]. A line of evidence has demonstrated that CR acts as a modulator of XREs and up-regulation of the expression of enzymes involved in phase I, phase II metabolism and xenobiotic transport in mouse liver [30, 61, 65]. CR led to an increase in microsomal enzyme activity of cytochrome P450 (CYP450), modifying phase I metabolism of environmental toxicants [31, 32]. Notably, CR enhanced detoxification of environmental chemicals and reduced the formation of adducts through increasing the activity of phase II metabolizing enzymes, such as glutathione S-transferases (GSTs), uridine diphosphate glucuronosyltransferase (UGTs), and NAD(P)H: quinone oxidoreductase (NQOs) [33, 66, 67]. In addition to the changes in enzyme activity described previously, we also identified that AhR- and Nrf2-mediated metabolism, glutathione-mediated detoxification, and RXR activation might be involved in the metabolism of PM-bound chemicals in mouse liver, thus determining the intensity of systemic toxicity. Notably, the urinary metabolites of PM-bound PAHs were positively associated with the expression of key metabolizing enzymes and decreased cytotoxicity in CR-fed mice, indicating that the clearance of toxic chemicals in CR-fed mice was greatly accelerated.

Based on transcriptome data, we reveal that the changes in the pathways induced by CR diet are tissue specific. CR mainly inhibited the inflammatory related pathways in lung but activated the pathways of xenobiotic metabolism in liver. The variety in pathway regulations were consistent with the biological functions and the response to PM exposure [8]. However, compatible pathway regulations could be shared by lung and liver of CR-fed mice, such as LXR/RXR, nicotine degradation pathways of xenobiotic metabolism. Moreover, PM-bound chemical components could be metabolically activated by enzymes highly induced in both lung and liver. Further study is required to address the correlation between the PM-bound chemical components and the toxicity pathways affected by CR in a tissue-specific manner.

## Conclusion

In conclusion, we demonstrated that CR has protective effects against PM-induced pulmonary injury and extra-pulmonary toxicity by reducing oxidative stress, DNA damage, and inflammatory responses. In addition, CR activates pathways involved in metabolic activation and detoxification of xenobiotics, facilitating the elimination of PM-bound toxic components, such as PAHs. These novel findings provide strong evidence for the significance and necessity of dietary interventions to reduce the health risk in PM-exposure population.

## Methods

### Animal models

Six-week-old male C57BL/6 mice were purchased from the Model Animal Research Center of Nanjing University (Nanjing, Jiangsu, China.). Mice were housed in cages under a 12-h dark/light cycle with free access to food and water. As shown in **Fig. S****1**, after 2-week acclimation period, mice were divided into two weight-matched groups and fed with different diets, including ad libitum diet (AL, *n* = 40) and caloric restriction diet (CR, *n* = 40) for a period of 4 weeks, respectively. CR was progressive, initiated at 10% restriction during the first week, 25% during the second week, and to 40% for the remainder experimental period, according to the method described previously [48, 68, 69]. The components of AL diet and CR diet are shown in **Table S****1**. The food intake and the water consumption were monitored daily (**Fig. S****2**) and the body weights were recorded weekly (Fig. 1a). All animal procedures were conducted in accordance with the guidelines of the Animal Care and Protection Committee of Sun Yat-sen University and Hebei Medical University.

### Real-ambient PM exposure

Mice were kept in isolated ventilated cages (IVC) and exposed to PM in a real-ambient PM exposure system located at the heart of city Shijiazhuang, China, where the annual average concentration of PM2.5 was ranked in the top 5 Chinese cities over the past decade. The characteristics of the real-ambient PM exposure system has been described in our previous study [8]. As shown in **Fig. S****3**, this system permitted circulating the ambient air into the chambers in absent of concentrating the ambient PM. The air channels of the control chambers are equipped with a three-layer HEPA filter (air filter, AF), which provides an excellent barrier to block fine ambient PM. Both AF control and PM exposure system were equipped with an auto-temperature control module to keep a constant temperature of the input air. The meteorological condition inside the chambers was closely monitored to keep a relatively constant temperature, humidity, ventilation frequency, air-flow rate, and noise (**Table S****2**). We measured PM concentration in the chambers using an Aerosol Detector DUSTTRAKTM II and analyzed the particle size spectrum using an Aerodynamic Particle Sizer Spectrometer 3321 (TSI Incorporated, Shoreview, MN, USA). Two groups of male mice (*n* = 20/group, 5 mice/cage) fed with AL or CR diet were exposed to PM for 24 h per day, 7 days per week for 4 weeks, from Jan 4th to Feb 1st, 2018. The other two groups of male mice (*n* = 20/group, 5 mice/cage) were kept in the control AF chambers. At the end of the experiments, mice were sacrificed, and the biological samples were collected for further analyses as described below.

### PM collection, extraction, and components analysis

In the course of PM exposure (4 weeks), the ambient PM_2.5_ was collected onto Teflon filters daily at a flow rate of 1.05 m^3^/min using a High-Volume Air Samples (Thermo Fischer Scientific, Waltham, MA, USA) nearby the PM exposure system. The filters were combined for chemical analysis. Organic components were extracted by Soxhlet extraction for quantification of polycyclic aromatic hydrocarbons (PAHs), nitro- and alkyl- derivatives of PAHs, polychlorinated biphenyls (PCBs), and polychlorinated dibenzo-dioxins (PCDD). Water-soluble fractions were extracted by sonication for analyses of metals and anion species with inductively coupled plasma-mass spectrometry (ICP-MS; ELEMENT2; Thermo Finnegan, San Jose, CA, USA).

### Blood collection and tissue preparation

At the end of PM exposure, mice were anaesthetized with 100 mg/kg sodium pentobarbital. Mouse blood was collected into the EDTA-coated tubes (BD Biosciences, San Jose, California). 100 μL blood was subjected for comet assay. The plasma was isolated from blood by centrifugation at 450×g for 10 min at room temperature and stored at − 80 °C before use. After the blood were collected, lung tissues and liver tissues were rapidly harvested and weighed. Then the tissues were divided into two parts for fixation in 4% paraformaldehyde in PBS and snap freeze in liquid nitrogen and stored at − 80 °C, respectively.

### BALF analysis

Bronchoalveolar lavage fluid (BALF) was collected as described previously [8]. The cells and supernatant were separated by centrifugation of BALF at 400×g for 7 min at 4 °C. Total protein, lactate dehydrogenase (LDH) and albumin contents in BALF supernatant were determined by BCA Protein Assay Kit (Beyotime Biotech Inc., Nantong, China), LDH release assay kit (Promega, Corporation, Madison, WI, USA) and Albumin Assay kit (Nanjing Jiancheng Bioengineering Institute, China), respectively. The number of cells was determined with a cell counter (Beckman, Coulter, CA, USA). 1 × 10^4^ cells were spread on microscope slides, fixed with 96% ethanol and stained with May-Grünwald-Giemsa. The number of macrophages and polymorphonuclear neutrophils (PMNs) were counted under microscope (Leica, Germany).

### Histopathological analysis

The lung tissues were removed, washed with 0.1 μM phosphate buffered saline (PBS, pH 7.4), fixed in 4% formalin for 24 h at room temperature, dehydrated by graded ethanol, and embedded in paraffin. Tissue sections (5 μm) were made and stained with hematoxylin and eosin. The histological examination was performed under a light microscope. The histopathological analysis of lung injury was conducted quantitatively as described previously [70].

### TUNEL staining

The lung sections (5 μM) were stained via terminal-deoxynucleotidyl transferase-mediated nick end labeling (TUNEL) staining (Beyotime Biotech Inc., Nantong, China) to examine the apoptosis of cells in mouse lung according to the manufacturer’s instructions. Murine lung sections without any stimuli were incubated with 0.01 U/ul DNase I for 10 min at room temperature (RT) and were regarded as a positive control. For quantification of apoptotic cells, 20 random fields per section in each group were counted, and the average number of apoptosis per section was calculated.

### Detection of ROS in mouse lung

Reactive oxygen species (ROS) levels in the lung tissues were detected with the fluorescent probe DHE (dihydroethidium; Sigma, USA) [71]. Briefly, the frozen sections (5 μm) were incubated with 10 μM DHE at 37 °C for 30 min. The human bronchial epithelial cells (16HBE) treated with 100 μM H_2_O_2_ for 30 min were regarded as a positive control. The slides were viewed under a fluorescence microscope (Leica, Germany). The intensity of DHE was analyzed by ImageJ software.

### Immunohistochemistry of F4/80

F4/80, also called as EGF-like module-containing mucin-like hormone receptor-like 1 (EMR1), is a heavily glycosylated G-protein-coupled receptor and is a well-known biomarker for mouse macrophages [72]. F4/80 was detected by immunohistochemistry (IHC). Briefly, lung sections (5 μm) were mounted onto the slides, deparaffinized and rehydrated, and heated in 0.1 M citrate buffer (pH 5.8) for antigen retrieval. To inactive endogenous peroxidase, we incubated slides with 3% H_2_O_2_ at RT for 15 min. After blocking with 2% BSA for 30 min at 37 °C, the slides were incubated with primary antibodies against F4/80 (#30325; Cell Signaling Technology, MA, USA) overnight at 4 °C. After incubation with the corresponding secondary antibody for an additional 1 h at room temperature in dark, the slides incubated in 10 μg/ml 3, 3 N-diaminobenzidine tertrahydrochloride (DAB; Beyotime Biotech Inc., Nantong, China) for 10 min. The nuclei were counterstained with hematoxylin for 10 s. The monocytes (THP-1) treated with 100 ng/ml phorbol myristate acetate for 48 h were regarded as a positive control. Positive staining cells were counted in 20 random fields per section in each group by light microscopy (Leica, Germany) and the average number of positive cells per section was calculated.

### Immunofluorescence of γ-H2AX

The phosphorylation of the histone 2AX at ser139 (γH2AX), which is a marker of DNA double-strand breaks (DSB) [73], is assessed by immunofluorescence. In brief, lung sections (5 μm) were mounted onto the slides and subjected for deparaffinization, rehydration, antigen retrieval and inactivation of endogenous peroxidase as described above. After blocking with 2% BSA for 30 min at 37 °C, the slides were incubated with primary antibodies against γ-H2AX (#ab11174; Abcam, Cambridge, UK) followed by incubation for Alexafluor-488 conjugated secondary antibody (Thermo Fisher Scientific™, Waltham, MA, USA) and 4′,6-diamidino-2-phenylindole (DAPI) for 1 h at room temperature. Digital images were taken by a laser scanning confocal microscope (Leica, Germany). 16HBE cells treated with 5 μg/ml etoposide for 12 h were regarded as a positive control. Positive staining cells were counted in 20 random fields per section in each group and the average number of positive cells per section was calculated.

### Cytokine analysis

Cytokines in BALF and plasma, including interferon-γ (IFN-γ), tumor necrosis factor (TNF-α), interleukin-1 beta (IL-1β), interleukin-4 (IL-4), interleukin-6 (IL-6), interleukin-10 (IL-10), interleukin-12 (IL-12p70), and transforming growth factor beta (TGF-β1) were measured with an ELISA assay kit (R&D Systems, MN, USA). The z-score transformation was utilized to calculate the relative level of cytokines according the following equation: Z= $\frac{X-\overline{X}}{SD}$ ($\overline{X}$ is the mean value, SD is standard deviation).

### Cell culture

The neuroblastoma cells (Neuro-2A), monocytes (THP-1), liver hepatocellular carcinoma cell (HepG2), human embryonic kidney cells (HEK), colon carcinoma cells (HCT-116) were obtained from American Type Culture Collection (ATCC, Manassas, VA, USA). The human bronchial epithelial cells (16HBE) was a gift form Dr. D. C. Gruenert (University of California, San Francisco) [74]. HepG2, 16HBE, HEK, and HCT116 cells were cultured in Dulbecco’s Modified Eagle Medium (DMEM, Gibco, USA) supplemented with 10% fetal bovine serum (FBS, Gibco, USA). Neuro-2A and THP-1 were cultured in minimum Eagle’s medium (MEM, Gibco, USA) and Roswell Park Memorial Institute-1640 (RPMI-1640, Gibco, USA), respectively, supplemented with 10% FBS. Cells were incubated at 37 °C in a humidified chamber with 5% CO_2_.

### Ex vivo assays

Colorimetric cell viability assay (MTS; Promega Corporation, Madison, WI, USA) was used to determinate the effect of the plasma on cell viability of multiple cell lines, including Neuro-2A, THP1, HepG2, HEK, HCT116, 16HBE. We seeded the cells in a 96-well plate at a density of 3 × 10^3^ and cultured them in a medium containing 10% of fetal bovine serum (FBS) and 1:100 plasma isolated from the mouse blood for 48 h. At the end, 20 μL of MTS reagent were added to each well and incubated for 2 h. The absorbance at 490 nm was determined. The cell that not treated with the plasma from the mouse blood were regarded as a negative control. The cell viability was presented as fold changes relative to the cell viability of the negative control.

### Immunoblotting

Total cellular protein of liver tissues was extracted by RIPA lysis buffer (150 M of NaCl, 1% Triton X-100, 0.5% deoxycholate, 0.1% SDS, and 50 mM Tris (pH 7.4)) containing protease inhibitors. 20 μg soluble proteins were separated by 8 ~ 12% SDS-PAGE and were transferred onto a nitrocellulose membrane (Pall Corporation, NY, USA). After being blocked with 5% fat free milk, the membranes were incubated with primary antibodies against caspase3 (#9662), cleaved caspase 3 (#9664), CYP1A2 (#14719) (Cell Signaling Technology, MA, USA), CYP1A1 (#ab3568), CYP1B1 (#ab33586), GSTT1 (#ab199337), GSTM1 (#ab113432), UGT1A1 (#ab194697) (Abcam, Cambridge, UK), and β-actin (#60008; Proteintech Group, AL, USA). Immunolabeling was visualized with HRP-conjugated anti-rabbit IgG or anti-mouse IgG (Santa Cruz Biotechnology, CA, USA). The density of the specific bands was quantified using ImageJ software.

### RNA sequencing

For each group, we randomly selected 3 mice for conducting RNA sequencing of mouse lung or liver tissues, 12 mice in total from 4 groups of mice (AL-fed or CR-fed with or without PM exposure). The frozen tissues of lung and liver (approximately 5 mg) were subjected to RNA isolation using TRIzol reagent (Invitrogen, Carlsbad, CA, USA). Total RNA samples were applied for RNA sequencing (Beijing Genomics Institute in Shenzhen, China). Briefly, Oligo dT magnetic beads were employed to trap mRNAs with poly A tails, and the mRNAs were fragmented and reversely transcribed to double-stranded cDNA (dscDNA) by random primers. The cDNAs were ligated adaptors and subjected to amplification by PCR. The PCR products were then denatured, and single stranded PCR products were cyclized by splint oligos with DNA ligase to construct cDNA library. The sequencing was performed with BGISEQ-500 platform (Beijing Genomics Institute, Wuhan, China). To process sequences, the reads were filtered to obtained using SOAnuke (https://github.com/BGI-flexlab/SOAPnuke) [75]. The clean reads were aligned to a reference genome using Bowtie2 (http://bowtie-bio.sourceforge.net/Bowtie2/index.shtml) [76]. Then, the FPKM method was used to calculate the unigene expression level by RSEM (http://deweylab.biostat.wisc.edu/rsem/rsem-calculate-expression.html) [77, 78]. Differentially expressed genes (DEGs) were identified with the R package DEGseq [79]. DEGs were restricted, with the fold-change greater than 1.5 times, *P* value lesser than 0.05 as the thresholds, by performing pairwise comparisons of the gene expression profiles of different diets with or without PM exposure (“CR-AF vs. AL-AF”, and “CR-PM vs. AL-PM”). The pheatmap R package was used to draw a heatmap of DEGs. To annotate the biological of the DEGs, the canonical pathway analysis and the disease and function analysis were conducted by Ingenuity Pathway Analysis (IPA) software (Qiagen, Germany). Significant differences were defined as *P* value was less than 0.01 and the absolute value of the z score was greater than 2.

### Quantitative polymerase chain reaction (qPCR)

We conducted reverse transcription with an Advantage RT-for-PCR Kit (Takara, Tokyo, Japan), and performed quantitative realtime PCR (qRT-PCR) with a SYBR Green PCR Master Mix (Toyobo, Tokyo, Japan). β-Actin is served as an internal control. The 2^–ΔΔCt^ method was used to calculate the relative expression of mRNAs. The primers used for qPCR are shown in **Table S****3**.

### Examination of urinary OH-PAHs

Mouse urine collected by housing animals in the metabolic cages (Type Y-3101, Yuyan Instruments Co. Ltd., Shanghai, China) for 24 h at the end of PM exposure [80]. The urine was centrifuged at 1000×g for 10 min at 4 °C. The supernatant was filtered through 0.22 μM filter (Pall Corporation, USA). Concentration of seven hydroxylated metabolites of PAHs (OH-PAHs), including 1-OHNap, 2-OHNap, 1-OHPhe, 4-OHPhe, 9-OHPhe, 2-OHFlu, 1-OHPyr, in mouse urine were analyzed with a LC-20A high performance liquid chromatography system (HPLC; Shimadzu, Japan) coupled with a Q-Trap 5500 mass spectrometer (MS/MS; AB SCIEX, USA). Five isotopically labeled chemicals were used as internal standards: d8–2-OHNap d9–2-OHFlu, ^13^C6–4-OHPhe, ^13^C6–1-OHPyr. The concentration was calculated by extrapolating the peak area of the sample from standard sets. Urinary OH-PAHs concentrations were adjusted with the content of urinary creatinine (μg/g Cre).

### Statistical analysis

Data are shown as the mean ± S.D. All statistical analysis was performed with SPSS 22.0 statistical software (SPSS Inc., Chicago, IL, USA). Student’s t-test was applied to analyze the difference between two groups and one-way analysis of variance (ANOVA) followed by Bonferroni’s post hoc test was used for comparisons between multiple experimental groups. Differences were considered significant at *P* < 0.05.

## Supplementary information

**Additional file 1.** Supplementary information available online, including Supplementary Materials and Methods, Figure S1to S14 and Table S1 to S11.

## Data Availability

The datasets used and/or analyzed during the current study are available from the corresponding author on reasonable request.

## Abbreviations

PM: Particulate matter
CR: Caloric restriction
AL: Ad libitum
IPA: Ingenuity Pathway Analysis
MPPD: Multiple-Path Particle Dosimetry model
PAH: Polycyclic aromatic hydrocarbons
PCB: Polychlorinated biphenyl
PCDD: Polychlorinated dibenzo dioxins
BALF: Bronchoalveolar lavage fluid
LDH: Lactate dehydrogenase
TUNEL: Terminal-deoxynucleotidyl transferase-mediated nick end labeling
γH2AX: Phosphorylated form of a histone variant H2AX at Ser 139
PMN: Polymorphonuclear neutrophil
DEG: Differentially expressed genes
8-OHdG: 8-hydroxydeoxyguanosine
AhR: Aryl hydrocarbon receptor
Nrf2: Nuclear factor E2-related factor 2
GSH: Glutathione
RXR: Retinoic acid receptors
CYP450: Cytochrome P450
UGT: Uridine diphosphate glucuronosyltransferase
GST: Glutathione S-transferases
