# Supplementary material for: Caloric restriction attenuates C57BL/6 J mouse lung injury and extra-pulmonary toxicity induced by real ambient particulate matter exposure
Source: Part Fibre Toxicol. 2020 Jun 5;17:22. doi: 10.1186/s12989-020-00354-2 (PMC7275546; doi:10.1186/s12989-020-00354-2)
Supplement: Supplementary file 1 — Additional file 1. Supplementary information available online, including Supplementary Materials and Methods, Figure S1to S14 and Table S1 to S11. [file 12989_2020_354_MOESM1_ESM.docx]

**Supplementary materials**

**Caloric restriction attenuates C57BL/6J mouse lung injury and** **systemic toxicity induced by real ambient particulate matter exposure**

Daochuan Li^1,#^, Shen Chen^1,#^, Qiong Li^1,#^, Liping Chen^1^, Haiyan Zhang^1^, Huiyao Li^1^, Dianke Yu^2^, Rong Zhang^3^, Yujie Niu^3^, Shaoyou Lu^1^, Lizhu Ye^1^, Xiaowen Zeng^1^, Guanghui Dong^1^, Rui Chen^4^, Michael Aschner^5^, Yuxin Zheng^2^, Wen Chen^1,*^

^1^Department of Toxicology, School of Public Health, Sun Yat-sen University, Guangzhou 510080, China

^2^Department of Toxicology, School of Public Health, Qingdao University, Qingdao 266021, China

^3^Department of Toxicology, School of Public Health, Hebei Medical University, Shijiazhuang 050017, China

^4^Department of Toxicology and Sanitary Chemistry, School of Public Health, Capital Medical University, Beijing 100069, China

^5^ Department of Molecular Pharmacology, Albert Einstein College of Medicine, Forchheimer 209, 1300 Morris Park Avenue, Bronx, NY,10461, USA.

^#^These authors contributed equally to this work.

^*^Correspondence to Wen Chen, Department of Toxicology, School of Public Health, Sun Yat-sen University, 74 Zhongshan Road 2, Guangzhou 510080, China. Tel: +86 20 87330599; Email: [chenwen@mail.sysu.edu.cn](mailto:chenwen@mail.sysu.edu.cn).

**Supplementary** **Materials**

**Supplementary Methods**

**1. Estimation of the cumulative lung burden of PM2.5 exposure**

The cumulative lung burden of PM2.5 exposure was calculated using the following equation (S1):

Estimated cumulative lung burden=MV×T×CON×DF (S1)

where MV is the minute ventilation in the exposed mice (mL/min); T is the total exposure time (min); CON is the mean concentration of PM2.5 (mg/m^3^); DF is the pulmonary deposition fraction of PM2.5 (m^3^), which is estimated by the Multiple-Path Particle Dosimetry Model software (MPPD 3.04, <https://www.ara.com/products/multiple-path-particle-dosimetry-model-mppd-v-304>) [1, 2].

**2. MDA measurement**

The levels of malondialdehyde (MDA) in lung tissue, BALF supernatant and plasma were measured by thiobarbituric acid (TBA) reactivity using the commercial colorimetric MDA Assay Kit (Beyotime Biotech Inc., Nantong, China) according to the manufacturer’s instructions. The absorbances were measured at 532 nm. The amounts of MDA in samples were calculated according to the standard curve. The concentration of MDA was normalized to protein concentration and expressed as μmol/mg protein. 16HBE cells treated with or without 100 μM H_2_O_2_ for 6 h were regarded as a positive or a negative control.

**3. GSH detection**

The levels of reduced glutathione (GSH) in lung tissue, BALF supernatant and plasma were measured with GSH and GSSG Assay Kit (Beyotime Biotech Inc., Nantong, China) according to the manufacturer’s instructions. The total glutathione level was measured by the 5,5′-dithiobis (2-nitrobenzoic acid)-GSSG recycling assay. The absorbance was measured at 412 nm. The concentrations of total glutathione in samples were calculated according to the standard curve. After samples and standards were treated with 2-vinylpyridine to block GSH, the GSSG level was quantified similarly to the total glutathione. The concentration of reduced GSH were calculated by subtracting the amount of GSSG from the total glutathione. The results were normalized to protein concentration and expressed as nmol/mg protein. 16HBE cells treated with or without 100 μM H_2_O_2_ for 6 h were regarded as a positive or a negative control.

**4.** **Alkaline comet assay**

The mouse peripheral blood cells were collected at the end of experiment and subjected to the alkaline comet assay immediately, according to the protocol reported previously [3]. In brief, a 5μL peripheral blood sample were mixed with 20μL of 0.8% low melting point agarose at 37℃ and spread onto a CometAssay® HT 20-well slide (Trevigen). Alkaline lysis, electrophoresis, neutralization and fixation were conducted sequentially, and analyzed for PI staining. The slides were viewed under a fluorescence microscope (Nikon Eclipse Ti-E) and 150 cells were randomly selected per slide were scored by Comet Assay Software Project-1.2.2 (University of Wroclaw, Poland). Olive tail moment (OTM) was selected for indicating the degree of DNA damage. 16HBE cells exposed to ionizing radiation for 30 min or not were regarded as negative or positive control.

**5.** **Hematology measurement**

Cell counting of white blood cell (WBC) and its subsets (neutrophil, monocyte, lymphocyte) were analyzed by HEMAVET 950FS (Drew, USA) according to the manufacturer’s instructions.

**6. Blood biochemistry analysis**

The plasma levels of glutamic-pyruvic transaminase (ALT), glutamic oxalacetic transaminase (AST), total bilirubin (TBIL), total protein (TP), Albumin (ALB), globulin (GLO), creatinine (CRE), glucose (GLU), triglyceride (TG), cholesterol (CHOL), serum high-density lipoproteins cholesterol (HDL-C), and low-density lipoproteins cholesterol (LDL-C) were determined by a biochemistry kit (MNCHIP, Tianjin, China) using an automatic biochemical analyzer Pointcare M3 (MNCHIP, Tianjin, China).

**7. Measurement of ATP contents**

ATP contents in liver tissues were measured with an ATP Assay Kit (Promega Corporation, Madison, WI, USA) and analyzed with a Micro-plate Spectrophotometer (Promega, Madison, WI, USA). Results were expressed as nmol/mg protein.

**8. Analysis of urinary 8-OHdG**

To detect urinary 8-OHdG, 100 μg DNA was dissolved in 80 μL deionized water. 5 μL of [13C10, 15N5]-8-OHdG at a concentration of 740 μg/L was added into the DNA solution and served as an internal standard. The DNA samples were converted into a single-stranded DNA by incubation at 95℃ for 10 min and were digested to single nucleotide fragments by treatment of 5 unit of nuclease P1 for 1 h at 37℃. The mixture was subsequently dephosphorylated by incubation with 10 units of alkaline phosphatase for 1 h at 37℃. The supernatant was subjected to LC-MS/MS analysis after centrifugation at 15,000g for 15 min. The concentration of cellular 8-OHdG was calculated by extrapolating the peak area of the sample from a set of standards (0, 0.25, 0.5, 0.75, 1.5, 2, 4 μg/L). Urinary 8-OHdG concentrations were adjusted for urinary creatinine levels.

**9. Flow cytometry analysis**

1×10^6^ cells isolated from BALF were blocked with anti-mouse FcR antibody (CD16/CD32; Biolegend, USA) for 15 min at 4°C in FACS buffer (PBS with 2% FBS and 1 mM EDTA), subsequently cells were stained for 15 min at 4°C with Zombie NIR Fixable Viability Kit (Biolegend, USA) to discriminate dead cells, and then surface stained with antibodies for F4/80 (FITC; Biolegend, USA), CD11b (PerPC/Cy5.5; Biolegend, USA), CD11c (PE; Biolegend, USA), CD206 (APC; Biolegend, USA) or isotype control for 30 min at 4°C. Cells were washed three times with FACS buffer, fixed with the Fixation/Permeabilization buffer (eBioscience, USA) for 40 min at 4°C and washed three times in Permeabilization buffer. Cells were then resuspended in FACS buffer and analyzed by flow cytometry using CytoFlex (Beckman Coulter, USA).

**Supplementary figures**

**
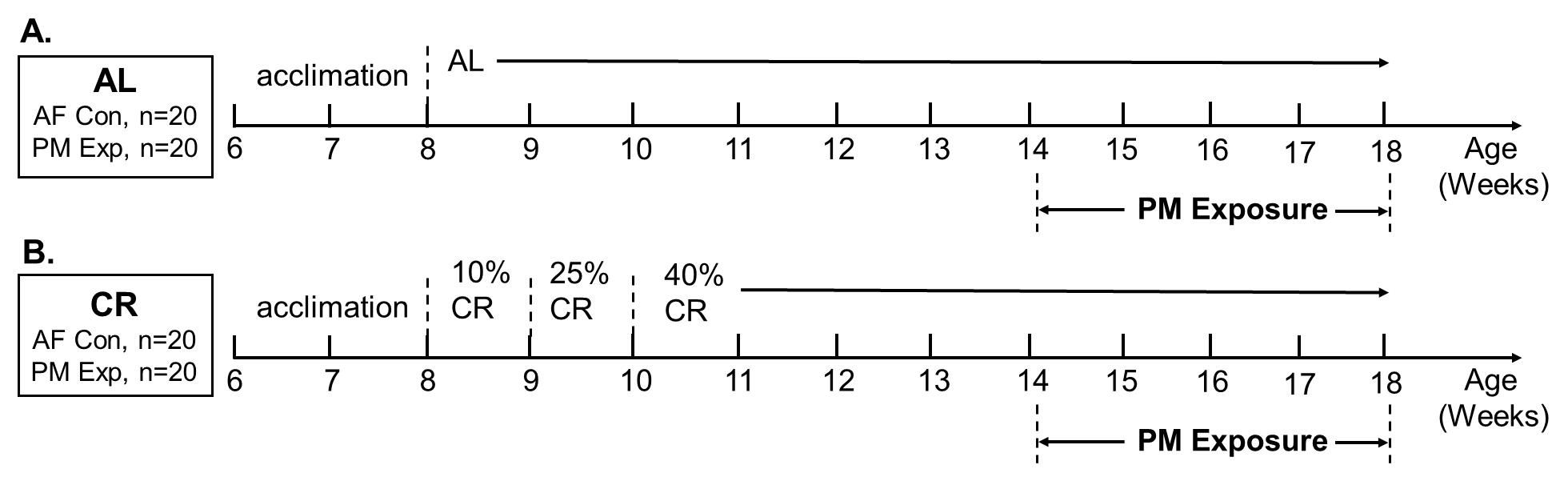
**

**Figure S1. The experiment design of caloric restriction and PM exposure.**

The diagram shows the design of the experiments of caloric restriction and PM exposure. Six-week-old C57BL/6 mice were acclimated for two weeks. The mice were then divided into two weight-matched groups and fed with different diets, including *ad libitum* diet (AL, n=40) (A) and caloric restriction diet (CR, n=40) (B) for a period of 4 weeks, respectively. To achieve the state of CR progressively, mice were fed with CR diet at 10% caloric restriction during the first week, 25% restriction during the second week, and up to 40% restriction for the subsequent experimental period. After adaption to 40% CR for four weeks, mice were exposed to ambient particulate matter (PM) for four weeks.


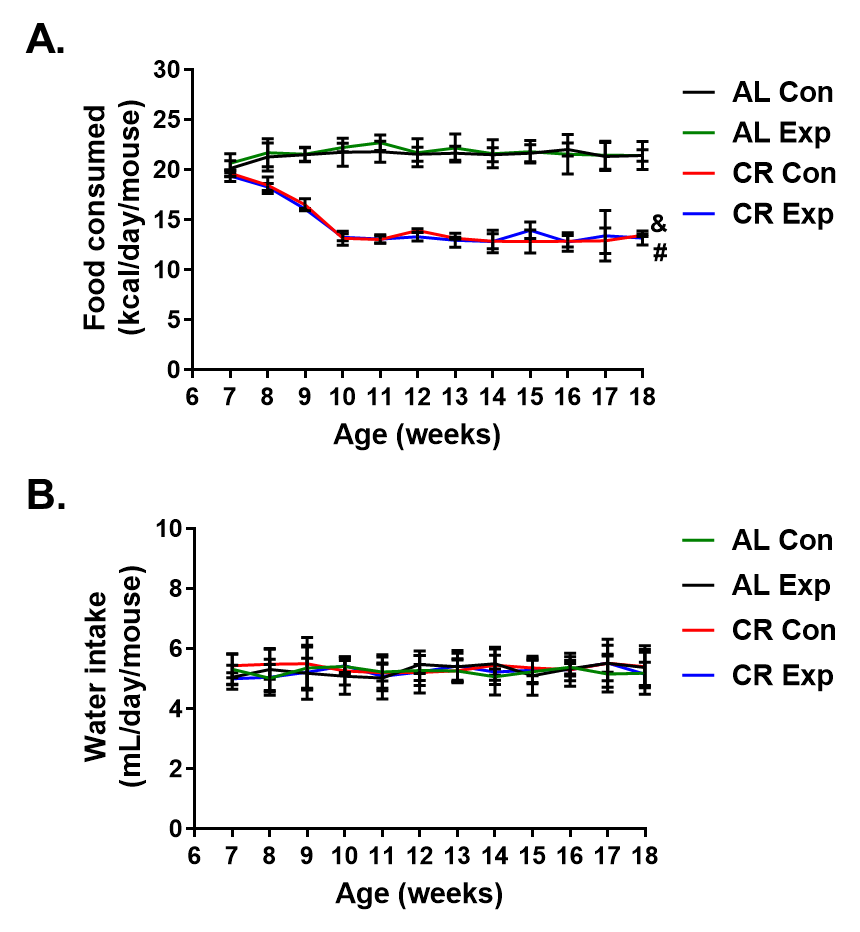


**Figure S2. Food intake of C57BL/6 J male mice fed with AL or CR diet.**

The data are expressed as the mean ± SD, & *P* < 0.05 CR-AF vs. AL-AF, # *P* < 0.05 CR- PM vs. AL- PM. n=20 per group.

**
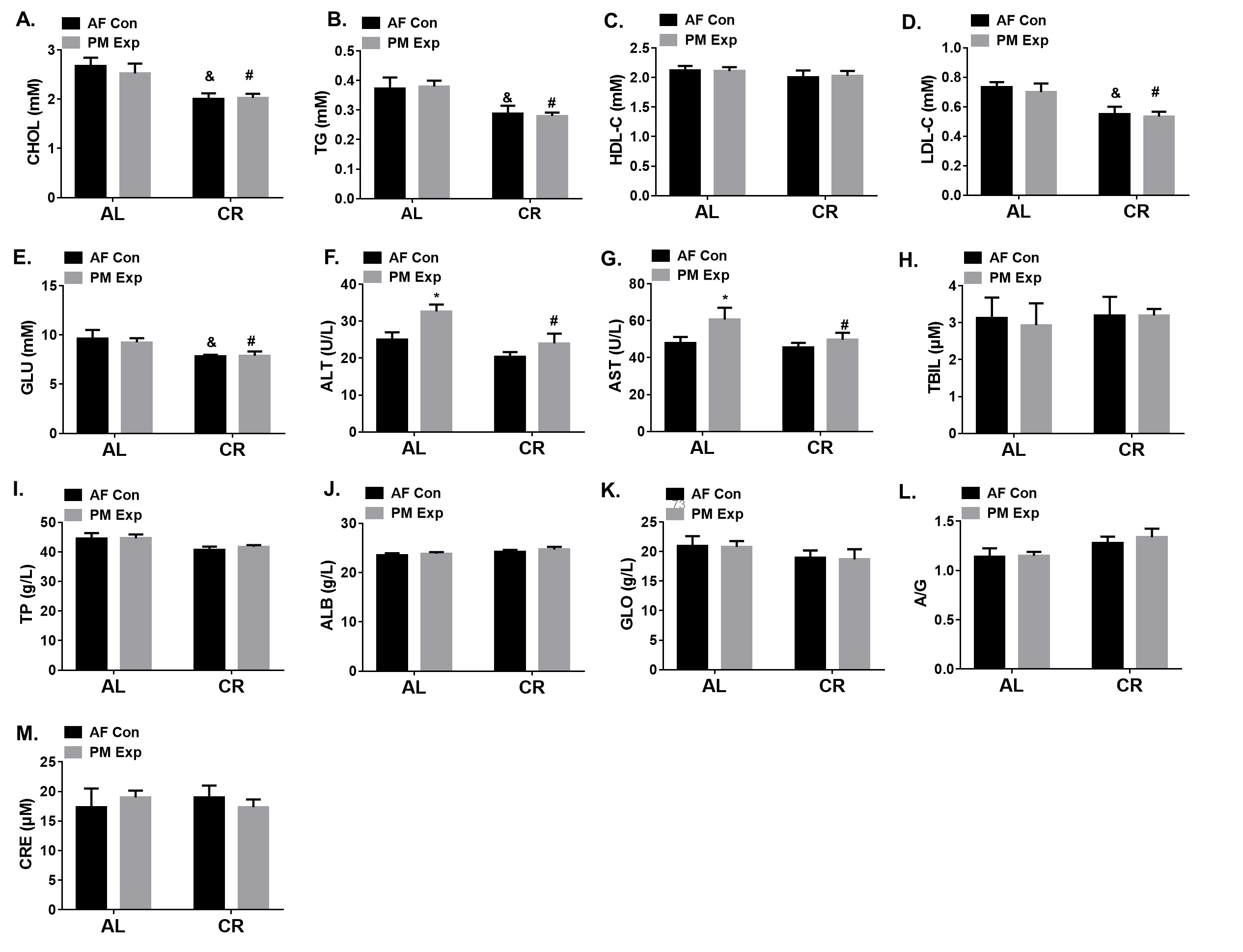
**

**Figure S3. Plasma biochemistry analysis.**

Plasma biochemistry analyses of (A) cholesterol (CHOL), (B) triglyceride (TG), (C) high-density lipoproteins cholesterol (HDL-C), (D) low-density lipoproteins cholesterol (LDL-C), (E) glucose (GLU), (F)glutamic-pyruvic transaminase (ALT), (G) glutamic-oxalacetic transaminase (AST), (H) total bilirubin (TBIL), (I) total protein (TP), (J) albumin (ALB), (K) globulin (GLO), (L) The ratio of ALB to GLO (A/G), and (M) creatinine (CRE). n=5 per group. The data are expressed as the mean ± SD, * *P* < 0.05 AF vs. PM, & *P* < 0.05 CR-AF vs. AL-AF, # *P* < 0.05 CR- PM vs. AL- PM.


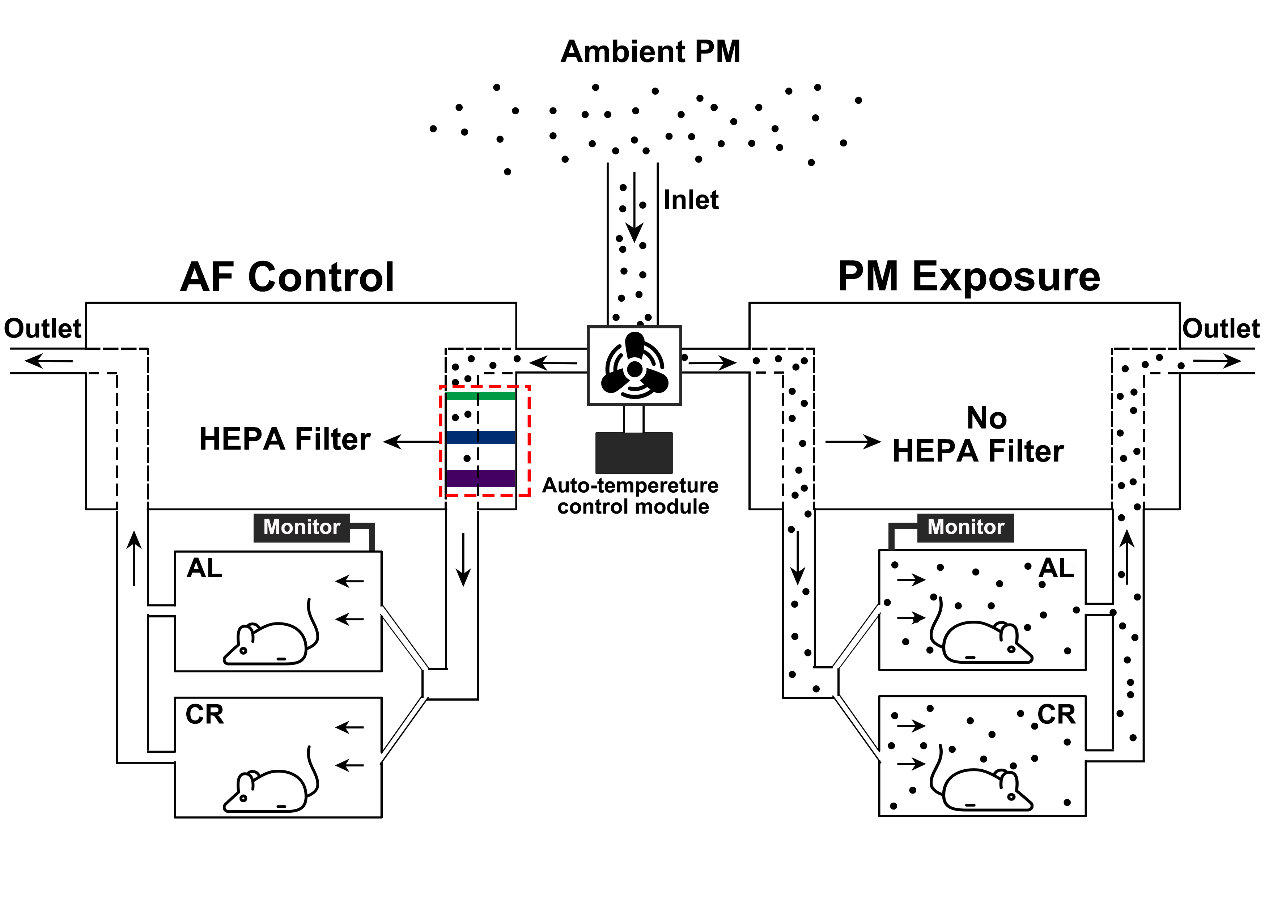


**Figure S4. The schematic diagram of a real-ambient PM exposure system.**

The ambient air was introduced into the AF control or PM exposure chambers through an air supply system with or without going through the HEPA filters as described previously [2]. The system was equipped with an auto-temperature control system to keep a constant temperature of the input air. The meteorological condition inside the chambers was closely monitored to keep a relatively constant temperature, humidity, ventilation frequency, air-flow rate, and noise.

**Figure S5. The concentration of PM2.5 in** **ambient air, PM exposure chamber, AF control chamber during exposure period.**

The concentration of PM_2.5_ was monitored in ambient air, PM exposure chamber, AF control chamber over the duration of the experiment. The dashed and dotted lines indicate the mean daily limit and a level of severe air pollution according to the Air Quality Guideline of China.

**Figure S6. The size distributions of particular matter (PM) in ambient air and PM exposure and AF control chambers.**

**
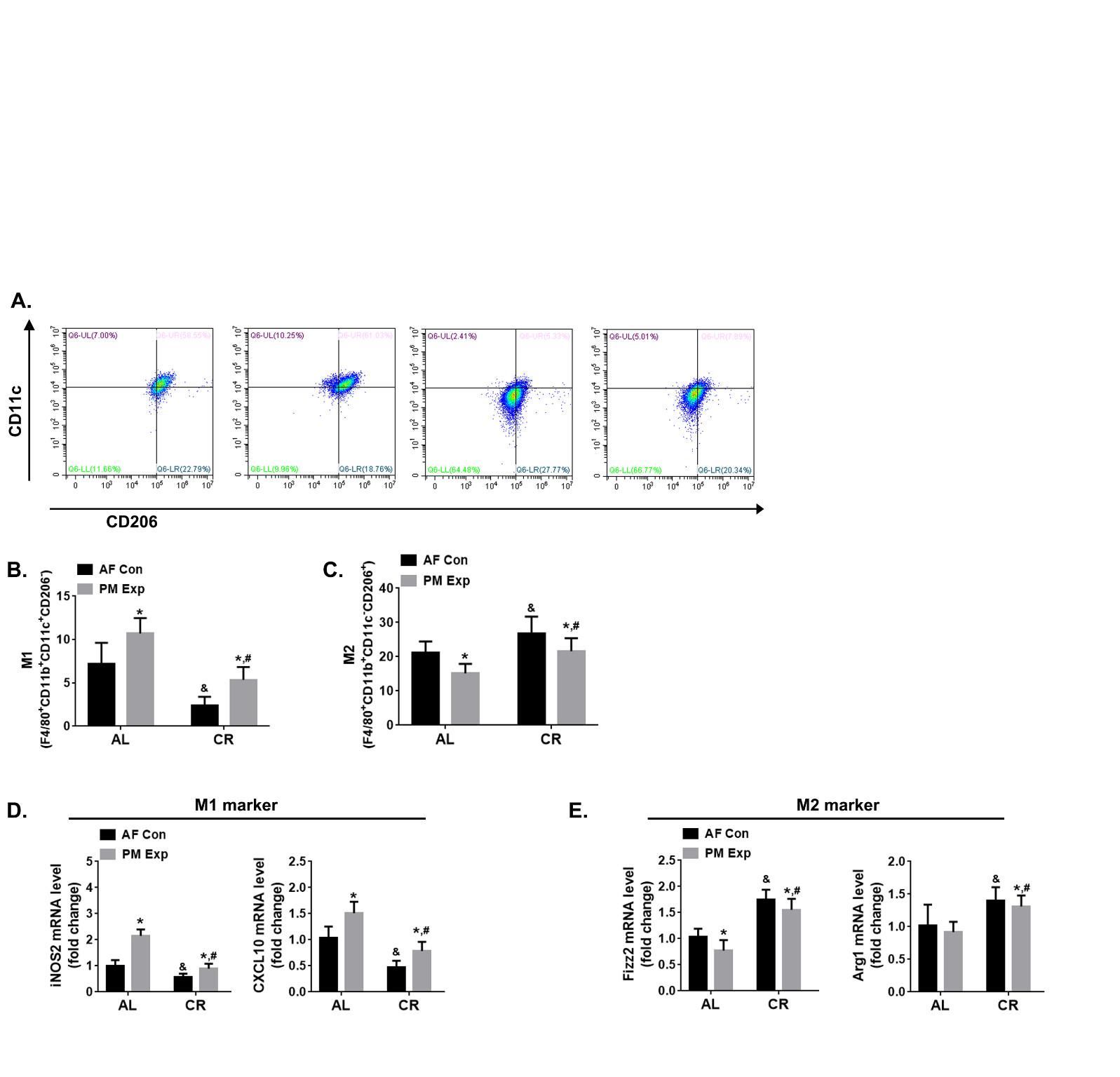
**

**Figure S7. CR inhibits M1 macrophage polarization and increases M2 macrophage polarization in response to PM exposure.**

(A) Flow cytometry analysis was conducted to examine the M1 and M2 macrophage polarization in BALF. The F4/80^+^/CD11b^+^/CD11c^+^/CD206^-^ macrophage was defined as M1 macrophage, and the F4/80^+^/CD11b^+^/CD11c^-^/CD206^+^ macrophage was defined as M2 macrophage. (B-C) The proportions of M1 and M2 macrophages in BALF. (D) The mRNA expression of M1 macrophage associated markers, *iNOS2* and *Cxcl10*. (E) The mRNA expression of M2 macrophage associated markers, *Fizz2* and *Arg1*. n=5 per group. The data are expressed as the mean ± SD, * *P* < 0.05 AF vs. PM, & *P* < 0.05 CR-AF vs. AL-AF, # *P* < 0.05 CR- PM vs. AL- PM.

**Figure S8. Analysis of the cytokine contents in BALF.**

The data are expressed as the mean ± SD, * *P* < 0.05 AF vs. PM, & *P* < 0.05 CR-AF vs. AL-AF, # *P* < 0.05 CR- PM vs. AL- PM. n=5 per group.

**
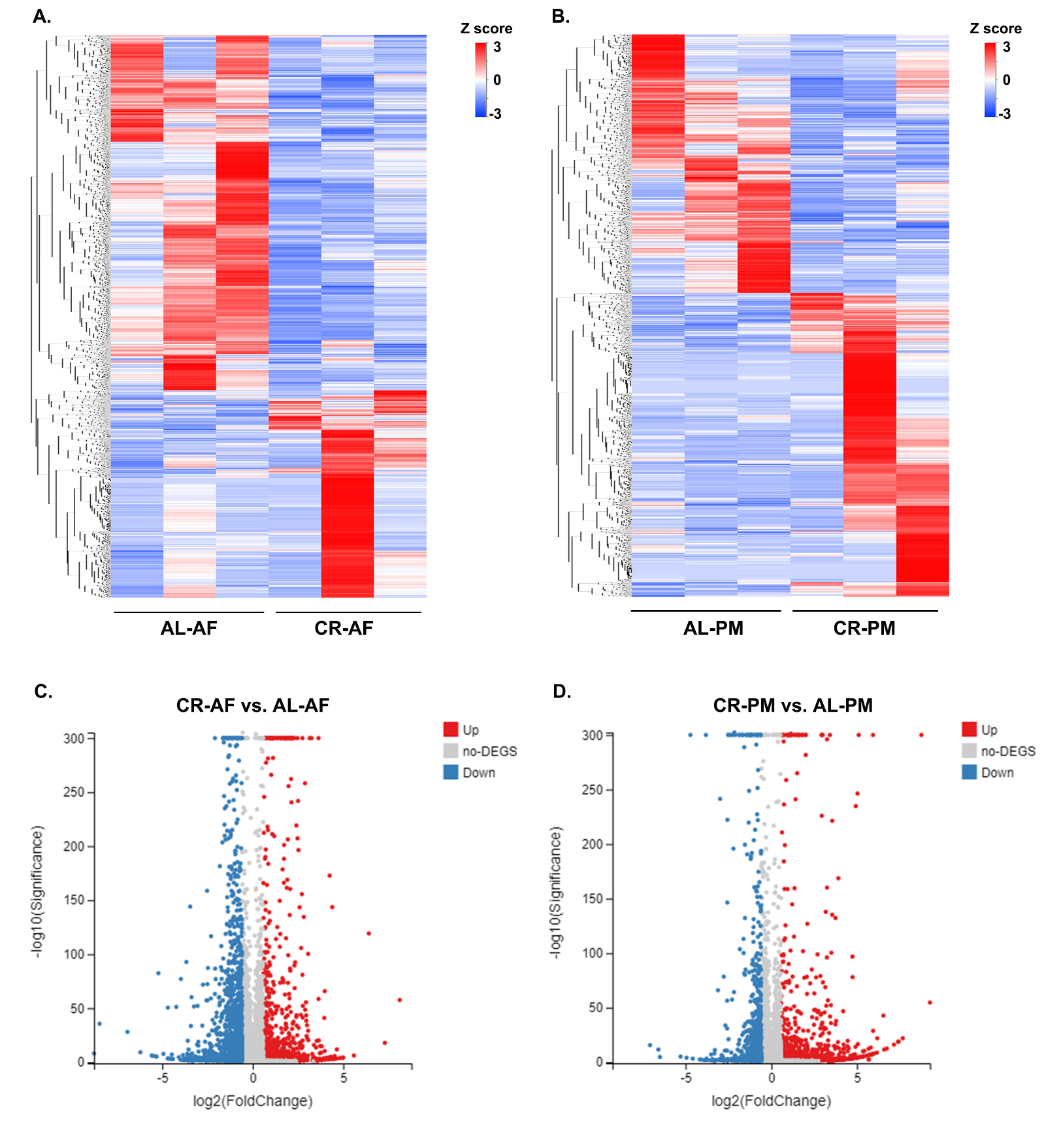
****Figure S9. The effects of CR on differential expressed genes (DEGs) in lung tissues of mice with or without PM exposure.**

(A-B) Transcriptomic profiles of lung tissues of AL-fed and CR-fed mice in AF control and PM exposure groups. The heatmap illustrates the expression of 2299 DEGs and 1928 DEGs in “CR-AF vs. AL-AF” and “CR-PM vs. AL-PM” comparisons, respectively. Average-linkage hierarchical clustering was performed using Pearson correlation. (C-D) Volcano plot of DEGs. The red and blue dots represented the up- and down-regulated genes, respectively. The black dots indicated the genes without significantly differential expression. n=3 per group.

**
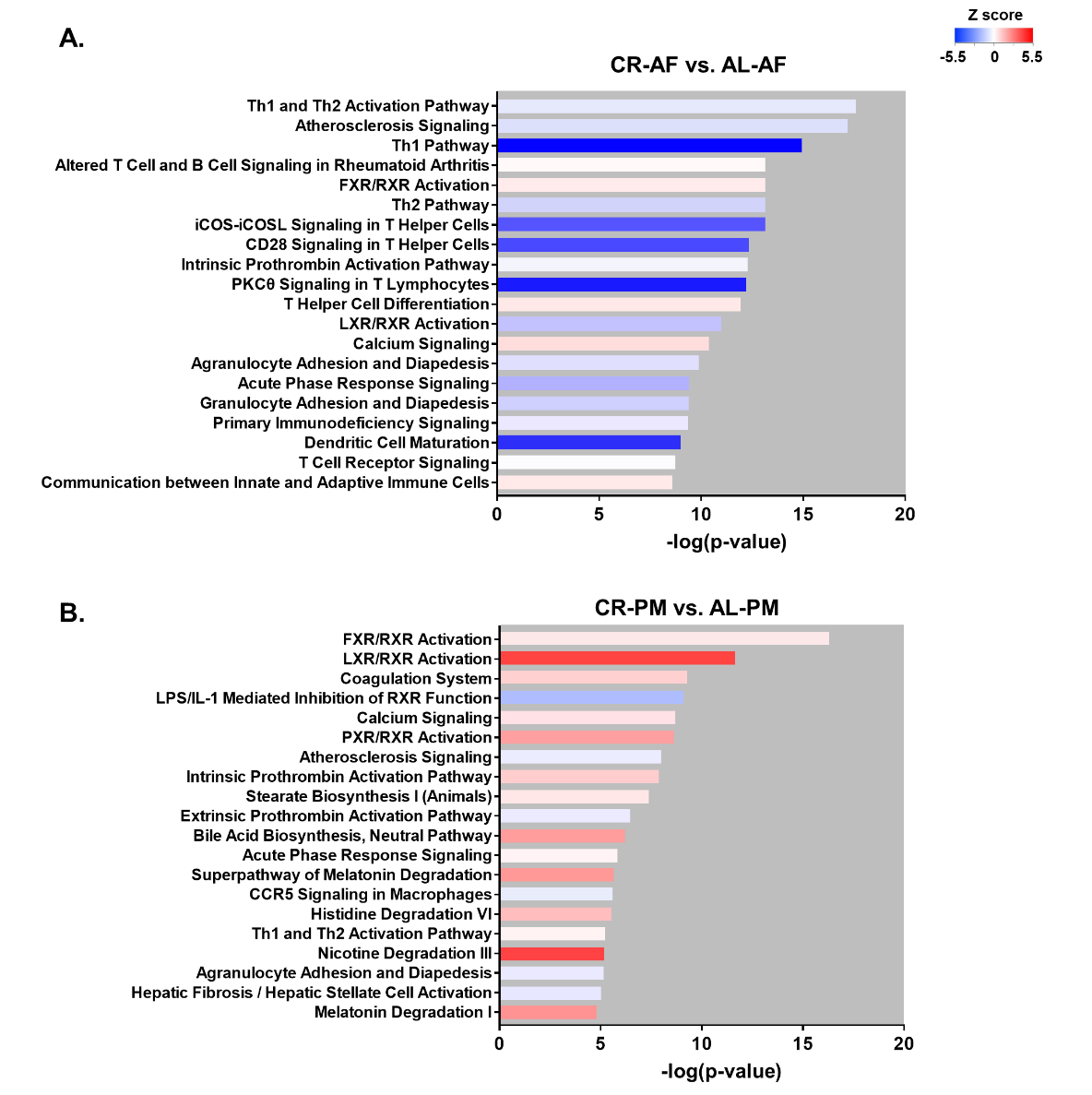
**

**Figure S10. Top 20 pathways in mouse lung.**

Comparison of the 20 most significant canonical pathways identified by IPA analysis between CR-AF and AL-AF mice (A) and between CR-PM and AL-PM mice (B).

**
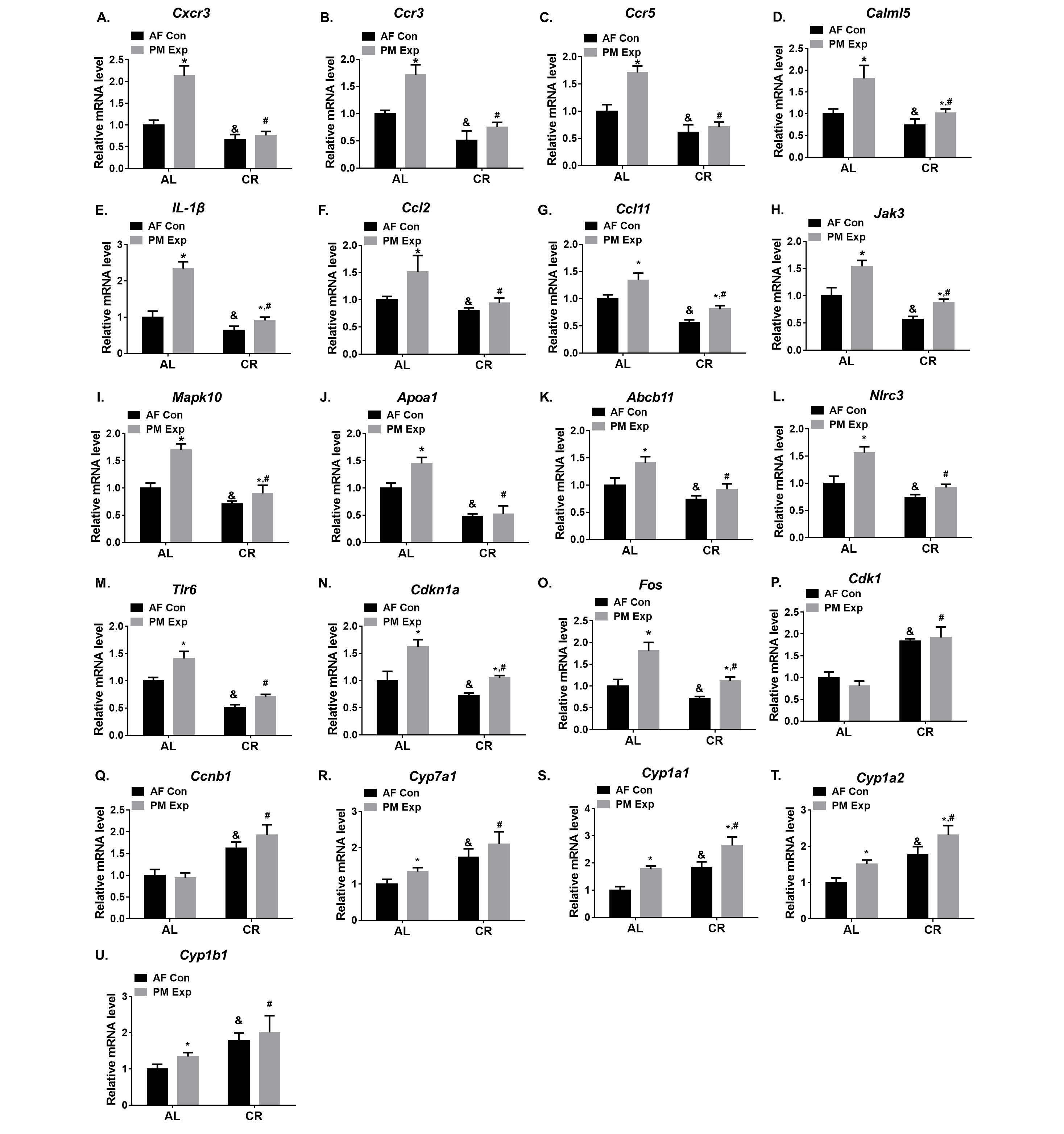
**

**Figure S11. Validation of the gene expression in mouse lung tissues.**

qPCR was performed for validation of the major genes involved in the regulation of key pathways. The data are expressed as the mean ± SD, * *P* < 0.05 AF vs. PM, & *P* < 0.05 CR-AF vs. AL-AF, # *P* < 0.05 CR- PM vs. AL- PM. n=3 per group.

**Figure S12. The levels of cytokines in plasma.**

The data are expressed as the mean ± SD, * *P* < 0.05 AF vs. PM, & *P* < 0.05 CR-AF vs. AL-AF, # *P* < 0.05 CR- PM vs. AL- PM. n=5 per group.


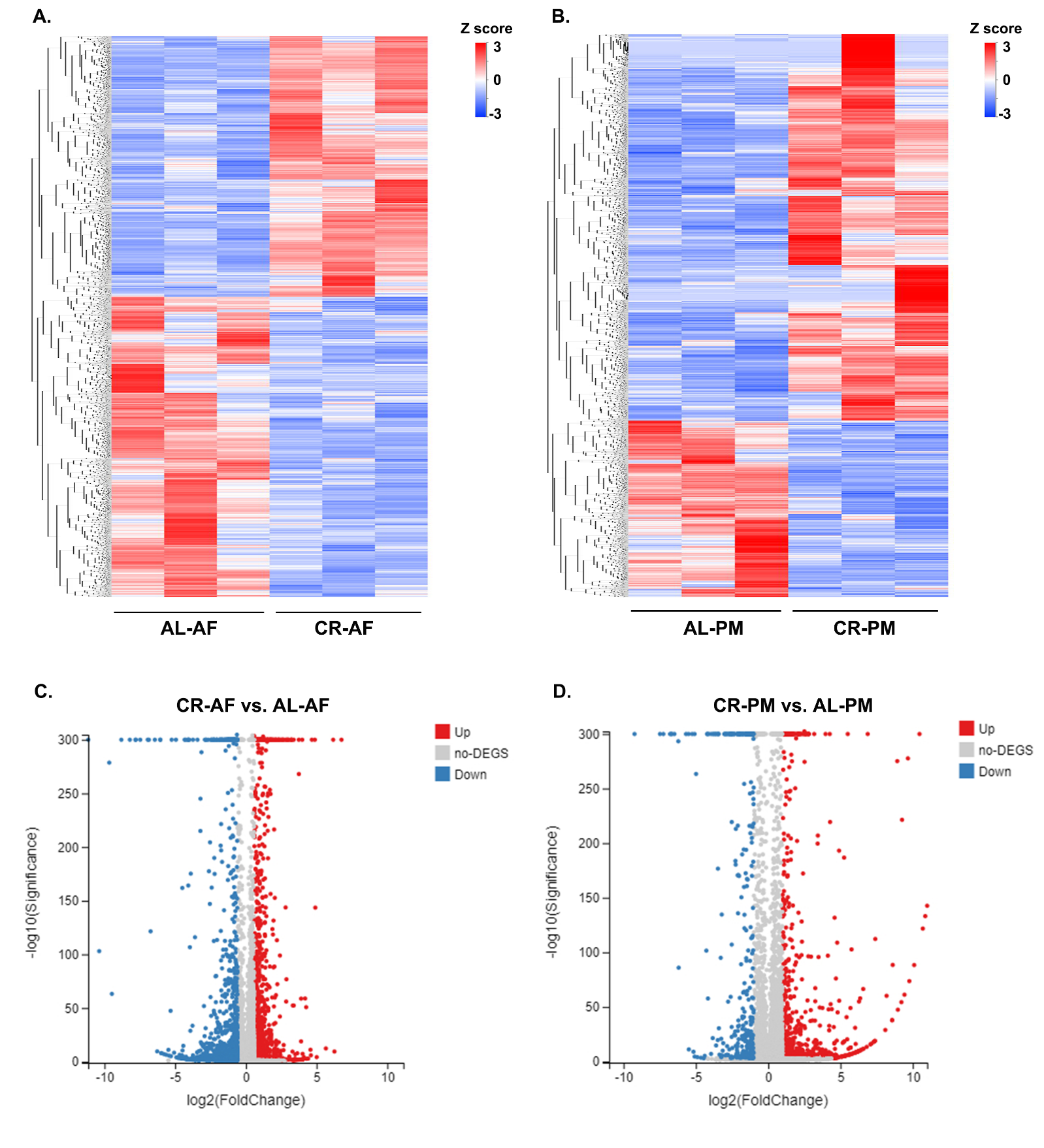


**Figure S13. The effects of CR on DEGs in liver tissues of mice with or without PM exposure group.**

(A-B) Transcriptomic profiles of liver tissues of AL-fed and CR-fed mice in AF control and PM exposure group. The heatmap illustrates expression of 2821 DEGs and 2995 DEGs in “CR-AF vs. AL-AF” and “CR-PM vs. AL-PM” comparisons, respectively. Average-linkage hierarchical clustering was performed using Pearson correlation. (C-D) Volcano plot of DEGs. The red and blue dots expressed the up- and down-regulated genes, respectively. The black dots indicated the genes without significantly differential expression. n=3 per group.

**
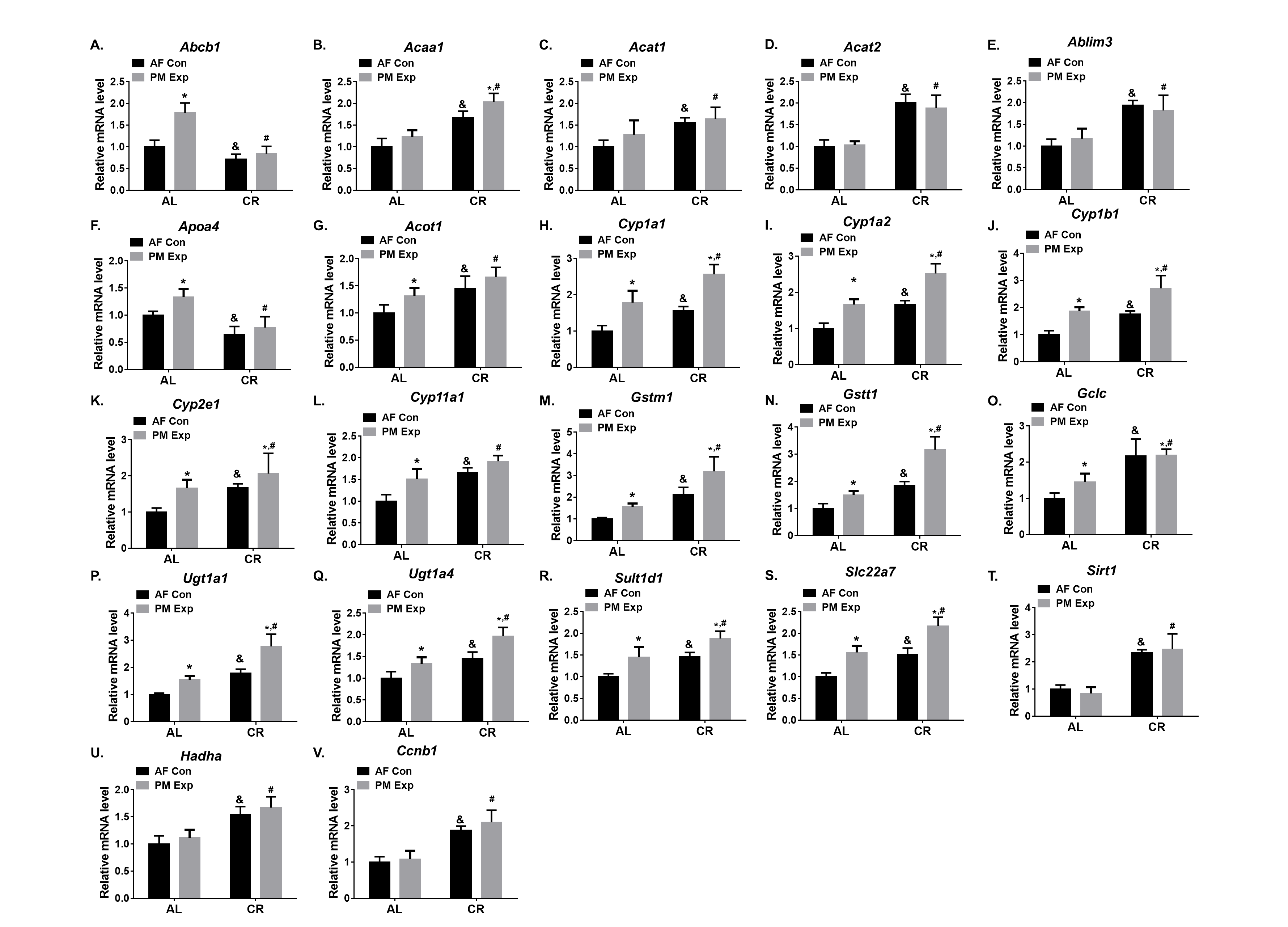
**

**Figure S14. Validation of the gene expression in mouse liver tissues by qPCR.**

qPCR was performed for validation of the major genes involved in the regulation of key pathways. The data are expressed as the mean ± SD, * *P* < 0.05 AF vs. PM, & *P* < 0.05 CR-AF vs. AL-AF, # *P* < 0.05 CR- PM vs. AL- PM. n=3 per group.

**Supplementary tables**

**Table S1. Characteristics of AL diet and CR diet [4].**

| **Ingredient** | **AL** | | **CR** | | | | | |
| --- | --- | --- | --- | --- | --- | --- | --- | --- |
|  |  |  | **-10%** | | **-25%** | | **-40%** | |
|  | gram | kcal | gram | kcal | gram | kcal | gram | kcal |
| Casein, 30 Mesh | 200.00 | 800.00 | 180.00 | 720.00 | 150.00 | 600.00 | 120.00 | 480.00 |
| L-Cystine | 3.00 | 12.00 | 2.70 | 10.80 | 2.25 | 9.00 | 1.80 | 7.20 |
| Corn Starch | 72.80 | 291.00 | 65.52 | 261.90 | 54.60 | 218.25 | 43.68 | 174.60 |
| Maltodextrin 10 | 100.00 | 400.00 | 90.00 | 360.00 | 75.00 | 300.00 | 60.00 | 240.00 |
| Sucrose | 172.80 | 691.00 | 155.52 | 621.90 | 129.60 | 518.25 | 103.68 | 414.60 |
| Soybean Oil | 25.00 | 225.00 | 22.50 | 202.50 | 18.75 | 168.75 | 15.00 | 135.00 |
| Lard | 177.50 | 1598.00 | 159.75 | 1438.20 | 133.13 | 1198.50 | 106.50 | 958.80 |
| Vitamin Mix V10001 | 10.00 | 40.00 | 10.00 | 40.00 | 10.00 | 40.00 | 10.00 | 40.00 |
| Mineral Mix S10026 | 10.00 | 0.00 | 10.00 | 0.00 | 10.00 | 0.00 | 10.00 | 0.00 |
| DiCalcium Phosphate | 13.00 | 0.00 | 13.00 | 0.00 | 13.00 | 0.00 | 13.00 | 0.00 |
| Calcium Carbonate | 5.50 | 0.00 | 5.50 | 0.00 | 5.50 | 0.00 | 5.50 | 0.00 |
| Potassium Citrate, 1H2O | 16.50 | 0.00 | 16.50 | 0.00 | 16.50 | 0.00 | 16.50 | 0.00 |
| Choline Bitartrate | 2.00 | 0.00 | 2.00 | 0.00 | 2.00 | 0.00 | 2.00 | 0.00 |
| Cellulose，BW200 | 50.00 | 0.00 | 125.11 | 0.00 | 242.77 | 0.00 | 358.44 | 0.00 |
| FD&C Red Dye #40 | 0.05 | 0.00 | 0.05 | 0.00 | 0.05 | 0.00 | 0.05 | 0.00 |
| Total | 858.15 | 4057.00 | 858.15 | 3651.30 | 858.15 | 3042.75 | 858.15 | 2434.20 |

**Table S2. The meteorological conditions in the AF control and PM exposure chambers**

|  | Temperature  (℃) | Humidity  （%） | Ventilation frequency  （/h) | Air-flow rate  （m/s) | Noise  （dB） |
| --- | --- | --- | --- | --- | --- |
| AF Control | 20.2±2.4 | 45.1±1.1% | 18-20 | 0.17 | 35-40 |
| PM Exposure | 21.5±3.1 | 46.3±2.1% | 18-20 | 0.17 | 35-40 |

**Table S3. Primers for qPCR.**

| **Gene name** | **Forward (5’-3’)** | **Reverse (5’-3’)** |
| --- | --- | --- |
| *iNOS2* | TCTCCCTTTCCTCCCTTCTT | CTTCAGTCAGGAGGTTGAGTTT |
| *Cxcl10* | TCAGGCTCGTCAGTTCTAAGT | CCTTGGGAAGATGGTGGTTAAG |
| *Fizz2* | TTCATCCTCGTCTCCCTTCT | TGAGAGCTTCCTTGATCCTTTG |
| *Arg1* | ACAGCAAAGCAGACAGAACTA | GAAAGGAACTGCTGGGATACA |
| *Cxcr3* | GTGCTTGTCCTCCTTGTAGTT | GTTGTCCTTGTTGCTGAGATTG |
| *Ccr3* | CCAGCTGTCAGCAGAGTAAA | CTCACCAACAAAGGCGTAGA |
| *Ccr5* | GCTCCAAGAGATGAGGAAAGAG | GAACACAGAGAGCAGTCGTTAT |
| *GATA4* | CAAAGGTGATAGACGGGAGAAG | GGTGATGAGGACAAGGAAGAAT |
| *IL-1β* | ATGGGCAACCACTTACCTATTT | GTTCTAGAGAGTGCTGCCTAATG |
| *Ccl2* | CTCGGACTGTGATGCCTTAAT | TGGATCCACACCTTGCATTTA |
| *Ccl11* | ACCCACTCTGCTCCCTATAA | CGTGAGCAGCAGGAATAGAA |
| *Jak3* | TGGCATCCTGCCTGTTTATC | TTGAGTGTCCACGTCCTCTA |
| *Mapk10* | CTGGCTTCTCTCAGTCAGTTATC | CTATGCTCCATGACCAAGTCTC |
| *Apoa1* | CTCTGGGTTCAACCGTTAGTC | TCTCCTGTCTCACCCAATCT |
| *Abcb11* | GCTCATCGCTTGTCTACTATCC | CCTTCTGGTCCATCAGTTTCTT |
| *Nlrc3* | GATGGCTTGGATGAGTGTAAGA | TTGCCTCGGATGATGTTAGTG |
| *Tlr6* | CAGAGTGAGTGGTGCCATTAT | GGTTCCAGCAAGATGAGGATTA |
| *Cdkn1a* | GTTCCTTGCCACTTCTTACCT | TCATCCTAGCTGGCCTTAGA |
| *Fos* | GAATCCGAAGGGAACGGAATAA | TCTCCGCTTGGAGTGTATCT |
| *Cdk1* | TTGAAGAGGCAACCGAGTAAG | GGCGTTAGGTCATCCATCAA |
| *Ccnb1* | GGTGTAACGGCCATGTTTATTG | CTGTCTGATCTGGTGCTTAGTG |
| *Cyp7a1* | CCACATCCTCCTGGCATTTA | CAAGAGCTGTAGACACTGAGAC |
| *Cyp1a1* | GTGAGCAAGGAGGCTAACTATC | GGCTACTGACACGACCAAATA |
| *Cyp1a2* | GTCTTCCTCTTCTTAGCCATCC | GCTTCATGGTCAACCCATAGT |
| *Cyp1b1* | TGGCCCTTTCCTCCTATCT | ACTGACACAACCTGCGTATC |
| *Abcb1* | AGGAGTCATCCTCTCCTTAGTC | GCCAGACAACAGCTTCATTTC |
| *Acaa1* | CAGGTTGTCACGCTACTCAA | TCAGTTCCCAGGGTATTCAAAG |
| *Acat1* | CTAAAGCCCTGGGTTCTTTCT | CTCGGTTCGTTTCTCTCTATGG |
| *Acat2* | AGCAGCATCCACGTACTTATTT | GTCAGTTCCAGTTCCAGTCATC |
| *Ablim3* | GCCTGTACTCTTCAGCCTTATT | GTTGCTTGCTGGAGCTTATTG |
| *Apoa4* | ACTGAGAGGGTGAAGGAAGA | CCGAACGTCTGGGTTACTTT |
| *Acot1* | CGTCATGGCTCTGGCTTATTA | CAGGTAGTTCACGGCTTCTT |
| *Cyp2e1* | GAAGTCTCTGGTTGACCCTAAG | AGGTCTCATGAACGAGGAATG |
| *Cyp11a1* | GGCATTTGAGGGTGGTATCT | GGAAAGAGGGAAAGAGGATGAA |
| *Gstm1* | CTCACGCTTCCTAGAATTACCC | CAGGCTGGCACTCAAGTATT |
| *Gstt1* | GAGTGTGGCTATCTTGCTCTAC | TGCCAGGTACTCGTCTACA |
| *Gclc* | CATCGACCTGACCATCGATAAG | AGGGTGAGTGGGTCTCTAATAA |
| *Sirt1* | GGAACCTTTGCCTCATCTACA | CACCTAGCCTATGACACAACTC |
| *Slc22a7* | TGTGTTCCGGGCATCATTAG | GCACAGATGGACAGGTACTTT |
| *Ugt1a1* | TGGACGGACTGCCTTTAATC | CAGCTAGGAGCATACTGGAATC |
| *B3gat3* | CCGGACAGAGAAACCTAAGATG | CCCACAAGGTATGTGCCTAAA |
| *Hadha* | AGACATCGGAGCTGTCTTTG | CACTACCTTCTGAGCACCATAC |
| *Sult1d1* | CAGTTCACTGTAGCCCAGTATG | CCAACTCTCCCTAGATCTCTGA |

**Table S4. Analysis of** **16 US EPA priority PAHs^#^ in the organic fraction of PM_2.5_.**

| **Components** | **TEF** | **μg/g PM** | **ng/m^3^** |  |
| --- | --- | --- | --- | --- |
|  |  |  |  |  |
| Naphthalene | 0.001 | BDL. | BDL. |  |
| Acenaphthylene | 0.001 | 5.50 | 0.83 |  |
| Acenaphthene | 0.001 | BDL. | BDL. |  |
| Fluorene | 0.001 | 4.78 | 0.72 |  |
| Phenanthrene | 0.001 | 60.78 | 9.20 |  |
| Anthracene | 0.01 | 6.82 | 1.03 |  |
| Fluoranthene | 0.001 | 153.81 | 23.29 |  |
| Pyrene | 0.001 | 123.63 | 18.72 |  |
| Benzo[a]anthracene | 0.1 | 66.87 | 10.13 |  |
| Chrysene | 0.01 | 101.69 | 15.40 |  |
| Benzo[b]fluoranthene | 0.1 | 183.37 | 27.77 |  |
| Benzo[ik]fluoranthene | 0.1 | 50.86 | 7.70 |  |
| Benzo[a]pyrene* | 1 | 67.43 | 10.21 |  |
| Indeno[1,2,3-cd]pyrene | 0.1 | 87.19 | 13.20 |  |
| Dibenzo[a,h]anthracene | 1 | 23.54 | 3.57 |  |
| Benzo[g,h,i]perylene | 0.01 | 81.16 | 12.29 |  |
| **𝛴_16_PAHs** |  | 1017.43 | 154.07 |  |
| **TEQs _PAH_** |  | 132.045 | 20.00 |  |

NOTE:

**^#^16 US EPA priority PAHs**: 16 PAHs from the USEPA priority list

**BDL**: below detection level.

*****The concentrations of benzo[a]pyrene in the Ambient Air Quality and Cleaner Air for Europe and

Air Quality Standards (GB 3095-2012) and Ministry of Ecology and Environment, China were

formulated as 1 ng/m^3^ and 2.5 ng/m^3^, respectively.

**TEF**: Toxic equivalent factor.

**Table S5. The analysis of 18 nitro-PAHs in the organic fraction of PM2.5.**

| **Components** | **TEF^a^** | **μg/g PM** | **ng/m^3^** |  |
| --- | --- | --- | --- | --- |
| 1-nitronaphthalene | — | 0.009 | 0.001 |  |
| 2-nitronaphthalene | — | 0.071 | 0.006 |  |
| 2-nitrobiphenyl | — | 0.001 | 0.000 |  |
| 3-nitrobiphenyl | — | 0.327 | 0.026 |  |
| 4-nitrobiphenyl | — | 0.168 | 0.014 |  |
| 1,3-dinitronaphthalene | — | 0.006 | BDL. |  |
| 3-nitrodibenzofuran | — | 0.095 | 0.008 |  |
| 2-nitrofluorene | 0.01 | 0.960 | 0.077 |  |
| 9-nitroanthracene | 0.0032 | 7.518 | 0.607 |  |
| 1,8-dinitronaphthalene | — | BDL. | BDL. |  |
| 9-nitrophenanthrene | — | 0.290 | 0.023 |  |
| 3-nitrophenanthrene | — | 1.281 | 0.103 |  |
| 2-nitroanthracene | — | 0.176 | 0.014 |  |
| 2-nitrofluoranthene | — | 8.169 | 0.660 |  |
| 1-nitropyrene | 0.1 | 0.461 | 0.037 |  |
| 2-nitropyrene | — | 3.272 | 0.264 |  |
| 7-nitrobenzo[a]anthracene | — | 1.320 | 0.107 |  |
| 6-nitrochrysene | 10 | 0.455 | 0.037 |  |
| **𝛴_18_nitro-PAHs** |  | 24.580 | 1.985 |  |
| **TEQs _nitro-PAH_** |  | 4.633 | 0.374 |  |

NOTE: ^a^TEF: Toxic equivalent factor.

**Table S6. Analysis of alkyl PAHs in the organic fraction of PM2.5.**

| **Components** | **μg/g PM** | **ng/m^3^** |
| --- | --- | --- |
| **Alkyl PAHs (μg/g PM)** |  |  |
| **PAH_166, methyl PAH166, DM PAH166** |  |  |
| fluorene | 5.3 | 0.51 |
| methyl fluorene-1 | 10.6 | 1.03 |
| methyl fluorene-2 | 2.6 | 0.25 |
| dimethyl fluorene-1 | 7.4 | 0.72 |
| dimethyl fluorene-2 | 9.4 | 0.91 |
| dimethyl fluorene-3 | 3.4 | 0.33 |
| dimethyl fluorene-4 | 1.5 | 0.14 |
| dimethyl fluorene-5 | BDL. | BDL. |
| dimethyl fluorene-6 | BDL. | BDL. |
| dimethyl fluorene-7 | BDL. | BDL. |
| **PAH_178, methyl PAH178, DM PAH178** |  |  |
| phenanthrene178 | 85.2 | 8.31 |
| anthracene178 | 50.3 | 4.90 |
| methyl PAH178_1 | 27.2 | 2.65 |
| methyl PAH178_2 | 55.6 | 5.42 |
| methyl PAH178_3 | 34.9 | 3.40 |
| methyl PAH178_4 | 51.9 | 5.05 |
| dimethyl-PAH178_1 | 14.6 | 1.42 |
| dimethyl-PAH178_2 | 19.2 | 1.87 |
| dimethyl-PAH178_3 | 30.8 | 3.00 |
| dimethyl-PAH178_4 | 67.5 | 6.58 |
| dimethyl-PAH178_5 | 13.7 | 1.33 |
| dimethyl-PAH178_6 | 7.1 | 0.69 |
| dimethyl-PAH178_7 | 9.1 | 0.89 |
| **PAH_202, methyl PAH202, DM PAH202** |  |  |
| fluoranthene202 | 261.3 | 25.47 |
| PAH202_3 | 23.2 | 2.26 |
| pyrene202 | 226.9 | 22.11 |
| methyl PAH202_1 | 46.8 | 4.56 |
| methyl PAH202_2 | 101.2 | 9.86 |
| methyl PAH202_3 | 66.6 | 6.49 |
| methyl PAH202_4 | 39.5 | 3.85 |
| methyl PAH202_5 | 25.2 | 2.45 |
| **PAH_226, methyl PAH226** |  |  |
| cyclopenta[cd]pyrene226 | 33.8 | 3.30 |
| methyl PAH226 | 8.5 | 0.83 |
| **PAH_228, methyl PAH228** |  |  |
| chrysene228 | 157.4 | 15.34 |
| triphenylene228 | 135.8 | 13.24 |
| methyl PAH228_1 | 26.9 | 2.62 |
| methyl PAH228_2 | 3.4 | 0.33 |
| methyl PAH228_3 | 50.5 | 4.92 |
| methyl PAH228_4 | 22.1 | 2.15 |
| methyl PAH228_5 | 9.7 | 0.94 |
| methyl PAH228_6 | 16.4 | 1.60 |
| **PAH_252, methyl PAH_252** |  |  |
| benzo[b+k]fluoranthene252 | 310.9 | 30.30 |
| benzo[a]fluoranthene252 | 29.9 | 2.91 |
| benzo[e]pyrene252 | 155.3 | 15.13 |
| benzo[a]pyrene252 | 179.9 | 17.53 |
| perylene252 | 24.0 | 2.34 |
| methyl PAH252_1 | 18.3 | 1.78 |
| methyl PAH252_2 | 24.0 | 2.34 |
| methyl PAH252_3 | 12.0 | 1.17 |
| methyl PAH252_4 | 25.1 | 2.45 |
| methyl PAH252_5 | 25.8 | 2.52 |
| methyl PAH252_6 | 22.6 | 2.20 |
| methyl PAH252_7 | 10.5 | 1.02 |
| **PAH_276, all unsubstituted** |  |  |
| PAH276_1 | BDL. | BDL. |
| PAH276_2 | 32.4 | 3.16 |
| indeno[1,2,3-cd]pyrene276 | 109.6 | 10.68 |
| benzo[ghi]perylene276 | 86.9 | 8.47 |
| **PAH_278, all unsubstituted** |  |  |
| PAH278_1 | 1.8 | 0.18 |
| PAH278_2 | 1.8 | 0.18 |
| PAH278_3 | 2.0 | 0.20 |
| PAH278_4 | 2.3 | 0.23 |
| PAH278_5 | 2.2 | 0.22 |
| PAH278_6 | 5.1 | 0.50 |
| PAH278_7 | 4.0 | 0.39 |
| PAH278_8 | 2.8 | 0.27 |
| PAH278_9 | 1.0 | 0.10 |
| PAH278_10 | 2.8 | 0.27 |
| PAH278_11 | 2.4 | 0.23 |
| dibenzo[a,h]anthracene | 12.6 | 1.22 |
| benzo[b]chrysene | BDL. | BDL. |
| dibenzo[a,c]anthracene | BDL. | BDL. |
| picene | BDL. | BDL. |
| **PAH_300** |  |  |
| Coronene | BDL. | BDL. |
| **𝛴 alkyl-PAHs** | 2870.50 | 279.71 |

NOTE: BDL: below detection level.

**Table S7. Analysis of 17 PCDD/F and 18 PCB in the organic fraction of PM2.5.**

| **Components** | **TEF** | **ng/g PM** | **pg/m^3^** | |  |
| --- | --- | --- | --- | --- | --- |
| **PCDD/F** | **I-TEF^a^** |  | |  |  |
| 2,3,7,8-TCDF | 0.1 | 1.884 | | 0.285 |  |
| 1,2,3,7,8-PeCDF | 0.05 | 2.493 | | 0.377 |  |
| 2,3,4,7,8-PeCDF | 0.5 | 2.884 | | 0.436 |  |
| 1,2,3,4,7,8-HxCDF | 0.1 | 4.536 | | 0.685 |  |
| 1,2,3,6,7,8-HxCDF | 0.1 | 3.436 | | 0.519 |  |
| 2,3,4,6,7,8-HxCDF | 0.1 | 5.182 | | 0.783 |  |
| 1,2,3,7,8,9-HxCDF | 0.1 | 0.565 | | 0.085 |  |
| 1,2,3,4,6,7,8-HpCDF | 0.01 | 9.895 | | 1.495 |  |
| 1,2,3,4,7,8,9-HpCDF | 0.01 | 1.915 | | 0.289 |  |
| **OCDF** | 0.001 | 13.016 | | 1.967 |  |
| 2,3,7,8-TCDD | 1 | 0.102 | | 0.015 |  |
| 1,2,3,7,8-PeCDD | 0.5 | 0.472 | | 0.071 |  |
| 1,2,3,4,7,8-HxCDD | 0.1 | 0.254 | | 0.038 |  |
| 1,2,3,6,7,8-HxCDD | 0.1 | 0.612 | | 0.093 |  |
| 1,2,3,7,8,9-HxCDD | 0.1 | 1.409 | | 0.213 |  |
| 1,2,3,4,6,7,8-HpCDD | 0.01 | 3.774 | | 0.570 |  |
| **OCDD** | 0.001 | 4.888 | | 0.739 |  |
| **𝛴 PCDF** |  | 45.806 | | 6.922 |  |
| **TEQs _PCDF_** |  | 3.258 | | 0.492 |  |
| **𝛴 PCDD** |  | 11.513 | | 1.740 |  |
| **TEQs _PCDD_** |  | 0.609 | | 0.092 |  |
| 𝛴 **PCDF+PCDD** |  | 57.318 | | 8.662 |  |
| **TEQs _PCDF+PCDD_** |  | 3.867 | | 0.584 |  |
|  |  |  | |  |  |
| **PCB** | **TEF_WHO-05_^b^** |  | |  |  |
| PCB 77 | 0.0001 | 2.323 | | 0.351 |  |
| PCB 81 | 0.0003 | 0.775 | | 0.117 |  |
| PCB 105 | 0.00003 | 1.808 | | 0.273 |  |
| PCB 114 | 0.00003 | 1.093 | | 0.165 |  |
| PCB 118 | 0.00003 | 3.427 | | 0.518 |  |
| PCB 123 | 0.00003 | 0.335 | | 0.051 |  |
| PCB 126 | 0.1 | 1.421 | | 0.215 |  |
| PCB 156 | 0.00003 | 1.511 | | 0.228 |  |
| PCB 157 | 0.00003 | 0.651 | | 0.098 |  |
| PCB 167 | 0.00003 | 0.649 | | 0.098 |  |
| PCB 169 | 0.03 | 0.715 | | 0.108 |  |
| PCB 189 | 0.00003 | 0.933 | | 0.141 |  |
| PCB 28 | — | 5.772 | | 0.872 |  |
| PCB 52 | — | 4.630 | | 0.700 |  |
| PCB 101 | — | 5.432 | | 2863.584 |  |
| PCB 138 | — | 2.995 | | 0.453 |  |
| PCB 153 | — | 2.806 | | 0.424 |  |
| PCB 180 | — | 3.100 | | 0.468 |  |
| **𝛴 PCB** |  | 40.375 | | 6.101 |  |
| **TEQs _PCB_** |  | 0.164 | | 0.025 |  |

Note: ^a^International Toxicity Equivalency Factor (I-TEF) for PCDD/F; ^b^The 2005 World Health

Organization reevaluation of human and mammalian toxic equivalency factors (TEFWHO-05) for PCB.

**Table S8. Analysis of elements and ion species in water-soluble components of PM2.5.**

| **Components** | **μg/g PM** | **ng/m^3^** |  |
| --- | --- | --- | --- |
| **Elements** |  |  |  |
| Li | 8.615 | 1.302 |  |
| Be | 0.036 | 0.005 |  |
| Na | 4145.329 | 626.379 |  |
| Mg | 1100.435 | 166.281 |  |
| Al | 147.722 | 22.321 |  |
| K | 6449.476 | 974.547 |  |
| Ca | 10633.979 | 1606.846 |  |
| Ti | 6.200 | 0.937 |  |
| V | 3.272 | 0.494 |  |
| Cr | 5.203 | 0.786 |  |
| Mn | 176.533 | 26.675 |  |
| Fe | 233.905 | 35.344 |  |
| Co | 1.153 | 0.174 |  |
| Ni | 2.889 | 0.436 |  |
| Cu | 37.322 | 5.640 |  |
| Zn | 448.365 | 67.750 |  |
| Ga | 4.765 | 0.720 |  |
| As | 23.878 | 3.608 |  |
| Se | 18.636 | 2.816 |  |
| Rb | 12.030 | 1.818 |  |
| Sr | 56.980 | 8.610 |  |
| Y | 0.379 | 0.057 |  |
| Zr | 1.564 | 0.236 |  |
| Nb | 0.039 | 0.006 |  |
| Mo | 7.343 | 1.110 |  |
| Pd | 0.066 | 0.010 |  |
| Ag | BDL. | BDL. |  |
| Cd | 2.980 | 0.450 |  |
| Sn | 5.118 | 0.773 |  |
| Sb | 17.454 | 2.637 |  |
| Te | 0.185 | 0.028 |  |
| Ba | 58.405 | 8.825 |  |
| Ta | BDL. | N.D. |  |
| W | 1.354 | 0.205 |  |
| Tl | 2.170 | 0.328 |  |
| Pb | 30.050 | 4.541 |  |
| **𝛴 Metal** | 23643.83 | 3572.70 |  |
| **Anions** |  |  |  |
| F^-^ | 1157.040 | 174.834 |  |
| Cl^-^ | 28279.831 | 4273.221 |  |
| SO_4_^2-^ | 72799.701 | 11000.391 |  |
| NO_3_^-^ | 104288.474 | 15758.498 |  |
| PO_4_^-^ | BDL. | BDL. |  |
| NO^2-^ | BDL. | BDL. |  |
| Br^-^ | BDL. | BDL. |  |
| **𝛴 Anions** | 206525.046 | 31206.944 |  |

**Table S9. Ambient air quality standards for different components in U.S. EPA, EU and China.**

|  | U.S. EPA^a^ | EU^b^ | China^c^ |
| --- | --- | --- | --- |
|  | ng/m^3^ | ng/m^3^ | ng/m^3^ |
| Benzo[a]pyrene | — | 1 | 2.5 |
| TEQs (PCDF+PCDD) | — | 0.1 | 0.1 |
| Cr | — | — | 000.025 (VI) |
| Ni | — | 20 | — |
| As | — | 6 | 6 |
| Pb | 0.15 | 0.5 | 1000 |
| Cd | — | 5 | 5 |
| F^-^ | — | — | 7000 |

Note: ^a^ Data were collected from National Ambient Air Quality Standards (NAAQS) established by U.S. EPA.

^b^ Data were collected from The Ambient Air Quality and Cleaner Air for Europe.

^c^ Data were collected from Air Quality Standards (GB 3095-2012) established by Ministry of Ecology and Environment of the People’s Republic of China.

**Table S10. The major DEGs in key canonical pathways involved in inflammatory response, oxidative stress, DNA damage, and xenobiotic metabolism in mouse lung.**

| **Ingenuity canonical pathways** | **Genes** |
| --- | --- |
| **CR-AF vs AL-AF** |  |
| **Inflammatory response** |  |
| Th1 pathway | CXCR3, CD247, CD274, CD28, CD3D, CD3E, CD3G, CD4, CD40LG, CD86, CD8A, CXCR3, H2-Eb2, HLA-A, HLA-DMB, HLA-DOA, HLA-DOB ,HLA-DQB1, HLA-DRB5, ICOS, ICOSLG/LOC102723996, CCR5, IFNG, IL10, IL10RA, IL12B, IL12RB1, IL18R1, IL27, IL27RA, JAK3, KLRC1, KLRD1, LTA, NFIL3, PIK3CD, PIK3CG, PIK3R5, PRKCQ, RUNX3, STAT4, TBX21, TNFSF11, VAV1 |
| Th2 pathway | CCR3, CCR1, CCR5, CCR8, CD247, CD28, CD3D, CD3E, CD3G, CD4, CD86, CXCR4, CXCR6, GFI1,H2-Eb2, HLA-A, HLA-DMB, HLA-DOA, HLA-DOB, HLA-DQB1, HLA-DRB5, ICOS, ICOSLG/LOC102723996, IFNG, IKZF1, IL10, IL12B, IL12RB1, IL2RB, IL2RG, IL4R, JAK3, PIK3CD, PIK3CG, PIK3R5, PRKCQ, RUNX3, SPI1, STAT4, TBX21, TNFRSF4, TNFSF4, VAV1 |
| Acute phase response signaling | IL-1β, A2M, ALB, AMBP, APCS, APOA1, APOA2, APOH, C1R, C5, C9, CRABP2, CRP, F2, F8, FGA, FGB, FGG, FOS, HPX,IKBKE, IL36A, IL36B, IL36RN, ITIH3, KLKB1, MAP3K1, NFKBID, NFKBIE, NGFR, OSM, PIK3CD, PIK3CG, PLG, RBP2, SAA1, SAA2-SAA4, Saa3, SERPINA1, SERPINA3, SERPIND1, SERPINF2, TNFRSF1B, TTR |
| CCR5 signaling in macrophages | CCR5, CACNA1B, CACNA1D, CACNA1E, CACNA1F, CACNA1I, CACNA1S, CACNA2D2, CACNG1, CACNG6, CACNG8, CALML5, CCL4, CCL5, CD247, CD3D, CD3E, CD3G, CD4, FASLG, FOS, GNG13, MAPK10, PLCG2, PRKCB, PRKCQ, PTK2B |
| Role of NFAT in Regulation of immune response | GATA4, BLNK, BTK, CALML5, CD247, CD28, CD3D, CD3E, CD3G, CD4, CD79A, CD79B, CD86, FCGR1A, FOS, GATA4, GNG13, H2-Eb2, HLA-A, HLA-DMB, HLA-DOA, HLA-DOB, HLA-DQB1, HLA-DRB5, IKBKE, ITK, LAT, LCK, LCP2, NFKBID, NFKBIE, PIK3CD, PIK3CG, PIK3R5,P LCB1, PLCG2, PRKCQ, SYK, ZAP70 |
| TREM1 signaling | NLRC3, CCL2, CD83, CD86, IL10, IL1B, LAT2, Naip1, NLRC4, NLRP10, NLRP12, NLRP3, NLRP6, PLCG2, TLR1, Tlr13, TLR6, TLR9 |
| Chemokine signaling | CCL11, CALML5, CAMK2A, CAMK2B, CCL2, CCL24, CCL4, CCL5, CCR3, CCR5, CXCR4, FOS, NOX1, PIK3CG, PLCB1, PLCG2, PRKCB, PTK2B |
| STAT3 pathway | CDKN1A, CSF2RB, CXCR2, IL10RA, IL11RA, IL12RB1, IL17RA, IL18R1, IL18RAP, IL1B, IL21R, IL22RA2, IL27RA, IL2RB, IL2RG, IL31RA, IL4R, IL5RA, IL9R, MAPK10, NGFR, NTRK1, PTPN6 |
| NF-κB signaling | TLR6, CARD11, CD40LG, IL1B, IL36A, IL36B, IL36RN, LCK, LTA, MALT1, MAP3K1, NFKBID, NFKBIE, NGFR, NTRK1, PIK3CD, PIK3CG, PIK3R5, PLCG2, PRKCB, PRKCQ, RELB, TLR1, TLR9, TNFRSF17, TNFRSF1B, TNFSF11, ZAP70 |
| HMGB1 signaling | CCL2, CD40LG, CLCF1, FASLG, FOS, IFNG, IL12B, IL1B, IL36A, IL36B, LTA, LTB, MAPK10, NGFR, OSM, PIK3CD, PIK3CG, PIK3R5, RAC2, RHOF, RHOH, TNFRSF1B, TNFSF11, TNFSF14, TNFSF4, Tnfsf9 |
| IL-17A signaling in airway cells | JAK3, CCL11, CCL20, CXCL6, IKBKE, IL17RA, MAPK10, MUC5AC, NFKBID, NFKBIE, PIK3CD, PIK3CG, PIK3R5 |
| **Oxidative stress** |  |
| Antioxidant action of vitamin C | MAPK10, CSF2RB, GSTO1, IKBKE, LCAT, NFKBID, NFKBIE, PLA2G2D, PLA2G2F, PLA2G3, PLA2G4C, PLA2G4D, PLA2G4E, PLA2G5, PLA2G7, PLAAT1, PLB1, PLCB1, PLCG2, PLCL2, PLD4, PNPLA3, SLC2A3, SLC2A4, SLC2A5 |
| Production of nitric oxide and reactive oxygen Species in macrophages | APOA1, ALB, APOA2, APOA4, APOB, APOC2, APOC4, APOF, FOS, IFNG, IKBKE, IRF8, JAK3, MAP3K1, MAPK10, NCF4, NFKBID, NFKBIE, NGFR, NOS2, PIK3CD, PIK3CG, PIK3R5, PLCG2, PPP1R3A, PPP1R3D, PRKCB, PRKCQ, PTPN6, RAC2, RHOF, RHOH, S100A8, SERPINA1, SPI1, TNFRSF1B |
| **DNA damage** |  |
| Cell cycle: G2/M DNA damage checkpoint regulation | CDK1, CCNB1, CCNB2, CDC25B, CDC25C, CDKN1A, CKS2, PKMYT1, PLK1, TOP2A, WEE1 |
| GADD45 signaling | CCNB1, CCNE1, CDK1, CDKN1A |
| **Xenobiotic metabolism** |  |
| LXR/RXR activation | CYP7A1, ACACA, ALB, AMBP ,APOA1, APOA2, APOA4, APOA5, APOB, APOC2, APOC4, APOF, APOH, C9, CCL2, FASN, FGA, GC, HPX, IL18RAP, IL1B, IL36A, IL36B, IL36RN, KNG1, LCAT, MLXIPL, MMP9, NGFR, NOS2, S100A8, SAA1, SCD, SERPINA1, SERPINF2, TNFRSF1B, TTR |
| LPS/IL-1 mediated inhibition of RXR function | ABCB11, ABCC2, ACSBG1, ACSL6, ALDH1L1, ALDH8A1, APOC2, APOC4, CHST10, CHST3,CYP2A12/CYP2A22, CYP2A6, CYP2C8, CYP2C9, CYP3A5, CYP3A7, CYP4A11, CYP4A14, CYP7A1, FABP1, FABP4, FABP5, FMO4, GSTA5, GSTO1, HMGCS2, HS3ST3A1, IL18RAP, IL1B, IL36A, IL36B, IL36RN, IL4I1, MAP3K1, NGFR, NR1I3, PPARGC1A, SLC10A1, SLC27A5, SLCO1A2, SLCO1B3, SULT1E1, TNFRSF1B |
| PXR/RXR activation | CYP1A1, ABCB11, ABCC2, CES3, CYP1A2, CYP1B1, CYP2A6, CYP2C8, CYP2C9, CYP3A5, CYP3A7, CYP7A1, G6PC, HMGCS2, HNF4A, IGFBP1, NR1I3, PPARGC1A, SCD, SLCO1B3, UGT1A1 |
| Nicotine degradation III | CYP1A2, ABCB11, ABCC2, CES3, CYP1A1, CYP1B1, CYP2A6, CYP2C8, CYP2C9, CYP3A5, CYP3A7, CYP7A1, G6PC, HMGCS2, HNF4A, IGFBP1, NR1I3, PPARGC1A, SCD, SLCO1B3, UGT1A1 |
| Nicotine degradation II | CYP1B1, ADH7, Aox4, CYP1A1, CYP1A2, CYP2A6, CYP2C40, CYP2C8, CYP2C9, CYP3A5, CYP3A7, FMO4, UGT1A1, UGT2B17, UGT2B28, UGT2B7, UGT3A1 |
| **CR-PM vs AL-PM** |  |
| **Inflammatory response** |  |
| Acute phase response signaling | IL-1β, AGT, AHSG, ALB, AMBP, APOA1, APOA2, APOH, C4A/C4B, C9, CRABP2, CRP, F2, FGA, FGB, FGG, FOS, HPX, ITIH3, MBL2, NGFR, PLG, RASD1, RASD2, RBP2, RBP7, SAA1, SAA2-SAA4, SAA3, SERPINA1, SERPINA3, SERPINF2, TTR |
| CCR5 signaling in macrophages | CCR5, CACNA1B, CACNA1G, CACNA1H, CACNA1I, CACNG1, CACNG2, CALML5, CCL5, CD247, CD3D, CD3E, CD3G, FAS, FASLG, FOS, GNG3, GNG4, MAPK10, OPN1SW, PRKCQ |
| Th2 pathway | CCR3, ACVR1C, CCR5, CD247, CD28, CD3D, CD3E, CD3G, DLL1, GFI1, HLA-DOB, HLA-DRA, IKZF1, IL13, IL2RA, IL2RB, IL4R, PRKCQ, PTGDR2, STAT4, TBX21, TNFRSF4, TSLP |
| Th1 pathway | CXCR3, CCR5, CD247, CD28, CD3D, CD3E, CD3G, CD40LG, CD8A, DLL1, HLA-DOB, HLA-DRA, IL27, KLRC1, KLRD1, NFIL3, PRKCQ, STAT4, TBX21 |
| STAT3 pathway | CDKN1A, IL11RA, IL13RA2, IL18RAP, IL1R2, IL20RA, IL20RB, IL22RA2, IL2RA, IL2RB, IL4R, IL5RA, MAPK10, NGFR, NTRK1, NTRK2, NTRK3, RASD1, RASD2 |
| Chemokine signaling | CCL11, CALML5, CAMK2A, CCL2, CCL24, CCL5, CCR3, CCR5, FOS, OPN1SW, RASD1, RASD2 |
| Role of NFAT in regulation of the immune response | GATA4, CALML5, CD247, CD28, CD3D, CD3E, CD3G, CD79A, CD79B, FCGR3A, FCGR3B, FOS, GNAT1, GNG3, GNG4, HLA-DOB, HLA-DRA, LAT, LCK, MEF2B, PRKCQ, RASD1, RASD2 |
| IL-12 signaling and production in macrophase | FOS, ALB, ALOX15, APOA1, APOA2, APOA4, APOB, APOC4, APOF, CD40LG, MAPK10, PRKCQ, SERPINA1, STAT4 |
| HMGB1 signaling | CCL2, CD40LG, CNTF, FASLG, FOS, IL11, IL13, IL17B, IL1B, LEP, MAPK10, NGFR, RAC2, RASD1, RASD2, RHOU |
| TREM1 signaling | NLRC3, CCL2, IL1B, MPO, NLRP12, NLRP6, TLR6 |
| NF-κB signaling | TLR6, CD40LG, IL1B, IL1R2, LCK, NGFR, NTRK1, NTRK2, NTRK3, PRKCQ, RASD1, RASD2, TNFRSF17 |
| **Oxidative stress** |  |
| Production of nitric oxide and reactive oxygen Species in macrophages | APOA1, ALB, APOA2, APOA4, APOB, APOC4, APOF, FOS, MAPK10, MPO, NGFR, PPARA, PPP1R3A, PPP1R3C, PRKCQ, RAC2, RHOU, SERPINA1 |
| Antioxidant action of vitamin C | MAPK10, LCAT, PLA2G2F, PLB1, PLD5, PLD6, PNPLA3, SLC2A2, SLC2A5, TXNDC2 |
| **DNA damage** |  |
| Cell cycle: G2/M DNA damage checkpoint regulation | CDK1, AURKA, CCNB1, CCNB2, CDC25C, CDKN1A, PLK1, SFN, TOP2A, WEE1 |
| GADD45 signaling | CCNB1, CDK1, CDKN1A, GADD45G |
| **Xenobiotic metabolism** |  |
| LXR/RXR activation | CYP7A1, ABCG8, AGT, AHSG, ALB, AMBP, APOA1, APOA2, APOA4, APOA5, APOB, APOC4, APOF, APOH, C4A, C4B, C9, CCL2, FASN, FGA, GC, HPX, IL18RAP, IL1B, IL1R2, IL1RAPL2, KNG1, LCAT, LPL, NGFR, RXRG, SAA1, SCD, SERPINA1, SERPINF2, TTR |
| LPS/IL-1 mediated inhibition of RXR function | ABCB11, ABCC2, ABCG8, ACSBG1, ACSL1, ALDH1L1, ALDH3A1, ALDH8A1, APOC4, CHST3, CPT1B, CYP2A12, CYP2A6, CYP2C8, CYP3A5, CYP4A11, CYP4A14, CYP7A1, FABP2, FABP3, FABP4, FABP5, FABP7, GAL3ST2, HMGCS1, HMGCS2, HS3ST4, IL18RAP, IL1B, IL1R2, IL1RAPL2, IL4I1, NGFR, NR0B2, NR1I3, PPARA, PPARGC1A, SLC10A1, SLC27A1, SLC27A2, SLC27A5, SLCO1A2, SLCO1B3, SULT1B1, SULT2A1 |
| PXR/RXR activation | CYP1A1, ABCB11, ABCC2, CES3, CYP1A2, CYP2A6, CYP2C8, CYP3A5, CYP7A1, G6PC, HMGCS2, HNF4A, IGFBP1, NR0B2, NR1I3, PPARA, PPARGC1A, PRKAR2B, SCD, SLCO1B3, SULT2A1, UGT1A1 |
| Nicotine degradation III | CYP1A2, AOX2, CYP1A1, CYP1B1, CYP2A6, CYP2C40, CYP2C8, CYP2E1, CYP3A5, UGT1A1, UGT2A3, UGT2B17, UGT2B28, UGT2B7, UGT3A1 |
| Nicotine degradation II | CYP1B1, AOX2, CYP1A1, CYP1A2, CYP2A6, CYP2C40 , CYP2C8, CYP2E1, CYP3A5, UGT1A1, UGT2A3, UGT2B17, UGT2B28, UGT2B7, UGT3A1 |

Note: Genes labeled in red were selected for validation of mRNA expression by qPCR (Fig.S12).

**Table S11. The major DEGs in the 20 most significant canonical pathways in mouse liver.**

| **Ingenuity canonical pathways** | **Genes** |
| --- | --- |
| **CR-AF vs AL-AF** |  |
| LPS/IL-1 Mediated Inhibition of RXR Function | ABCB1, ABCB11, ABCC2, ABCG5, ABCG8, ACOX1, ACOX2, ACSBG1, ACSL1, ACSL3, ALDH1A2, ALDH1L1, ALDH1L2, ALDH3A2, CES2, CHST1, CHST10, CHST11, CHST3, CHST4, CPT1A, CPT1B, CPT2, CYP2A6, CYP2C8, CYP4A11, CYP4A14, CYP7A1, FABP2, FABP5, FMO1, FMO2, FMO3, FMO4, FMO5, GSTA3, GSTA5, GSTM1, GSTM2, GSTM3, GSTM4, GSTM5, Gstm6, GSTT2, HMGCS2, HS3ST4, HS6ST1, IL18, IL18RAP, IL1A, IL1R1, IL1RL2, IL1RN, IL36G, JUN, MAOA, MAOB, MGST1, MGST3, NR0B2, PAPSS2, SLC27A1, SLC27A2, SREBF1, SULT1C2, Sult1D1, SULT1E1, TNFRSF1B |
| Superpathway of Cholesterol Biosynthesis | ACAT1, ACAT2, CYP51A1, DHCR7, FDFT1, FDPS, HADHA, HADHB, HMGCR, HMGCS2, IDI1, LBR, LSS, MVD, NSDHL, PMVK, SC5D, SQLE |
| Xenobiotic Metabolism Signaling | CYP1A1, ABCB1, ABCC2, ALDH1A2, ALDH1L1, ALDH1L2, ALDH3A2, ARNT, ATM, CAMK4, CES1, CES1G, CES2, CES3, CES4A, CHST1, CHST10, CHST11, CHST3, CHST4, CREBBP, CYP1A2, CYP1B1, CYP2C8, EIF2AK3, FMO1, FMO2, FMO3, FMO4, FMO5, FTL, GCLC, GSTA3, GSTA5, GSTM1, GSTM2, GSTM3, GSTM4, GSTM5, GSTM6, GSTT2, HDAC4, HS3ST4, HS6ST1, HSP90AA1, IL1A, MAOA, MAOB, MAP2K6, MAP3K13, MAPK12, MGST1, MGST3, NFE2L2, NQO1, NQO2, PIK3CG, PIK3R3, RASD1, RRAS, SULT1C2, SULT1D1, SULT1E1, UGT1A1, UGT1A3, UGT1A4, UGT1A7, UGT2A1, UGT2B17, UGT2B28, UGT2B7 |
| Nicotine Degradation II | CYP1A2, AOX1, B3GAT3, CYP1A1, CYP1B1, CYP2A6, CYP2C18, CYP2C40, CYP2C8, CYP2S1, CYP51A1, FMO1, FMO2, FMO3, FMO4, FMO5, INMT, UGT1A1, UGT1A3, UGT1A4, UGT1A7, UGT2A1, UGT2B17, UGT2B28, UGT2B7, UGT3A1 |
| Aryl Hydrocarbon Receptor Signaling | CYP1B1, ALDH1A2, ALDH1L1, ALDH1L2, ALDH3A2, ARNT, ATM, CCNA2, CCND1, CCND3, CCNE1, CCNE2, CDKN1A, CHEK1, CYP1A1, CYP1A2, DCT, E2F1, FAS, FOS, GSTA3, GSTA5, GSTM1, GSTM2, GSTM3, GSTM4, GSTM5, GSTM6, GSTT2, HSP90AA1, HSPB1, IL1A, JUN, MGST1, MGST3, NFE2L2, NQO1, NQO2, NR0B2, POLA1, TGFB3 |
| PXR/RXR Activation | GSTM1, ABCB1, ABCB11, ABCC2, ALDH3A2, CES2, CES3, CPT1A, CYP1A2, CYP2A6, CYP2C8, CYP7A1, FOXO1, GSTM2, HMGCS2, IGFBP1, NR0B2, PAPSS2, PCK2, PRKAR2B, SCD, UGT1A1, UGT1A7 |
| FXR/RXR Activation | SLC22A7, A1BG, ABCB11, ABCB4, ABCC2, ABCG5, ABCG8, ALB, APOA4, APOD, C3, C9, CREBBP, CYP7A1, CYP8B1, FASN, FOXA1, FOXO1, IL18, IL1A, IL1RN, IL36G, KNG1, MAPK12, MLXIPL, NR0B2, PCK2, PKLR, SAA1, SDC1, SERPINA1, SLC51B, SREBF1, TF, VLDLR |
| Fatty Acid β-oxidation I | ACAA1, ACAA1B, ACADM, ACSBG1, ACSL1, ACSL3, ECI1, EHHADH, HADHA, HADHB, HSD17B10, HSD17B4, SCP2, SLC27A1, SLC27A2 |
| LXR/RXR Activation | APOA4, A1BG, ABCG5, ABCG8, ACACA, ALB, APOD, C3, C9, CD36, CYP51A1, CYP7A1, FASN, FDFT1, HMGCR, IL18, IL18RAP, IL1A, IL1R1, IL1RL2, IL1RN, IL36G, KNG1, LDLR, MLXIPL, PTGS2, SAA1, SCD, SERPINA1, SREBF1, TF, TNFRSF1B, UGT1A3 |
| Superpathway of Melatonin Degradation | B3GAT3, CYP1A1, CYP1A2, CYP1B1, CYP2A6, CYP2C18, CYP2C40, CYP2C8, CYP2S1, CYP51A1, MAOA, MAOB, SULT1D1, UGT1A1, UGT1A3, UGT1A4, UGT1A7, UGT2A1, UGT2B17, UGT2B28, UGT2B7, UGT3A1 |
| Mevalonate Pathway I | ACAT1, ACAT2, HADHA, HADHB, HMGCR, HMGCS2, IDI1, MVD, PMVK |
| Agranulocyte Adhesion and Diapedesis | ACTA1, ACTA2, ACTG2, AOC3, C5AR1, CCL21, CCL4, CCL5, CCL8, CCL9, CD34, CKLF, CLDN1, CLDN18, CLDN2, CXCL10, CXCL11, CXCL9, CXCR1, CXCR4, GNAI1, IL18, IL1A, IL1R1, IL1RN, IL36G, ITGA4, MMP11, MMP15, MMP2, MMP23B, MMP27, MMP3, MMP7, MYH10, MYH11, MYH6, MYL6B, MYL9, PF4, PPBP, SELE, SELL, SELP, SELPLG |
| Nicotine Degradation III | UGT1A1, AOX1, B3GAT3, CYP1A1, CYP1A2, CYP1B1, CYP2A6, CYP2C18, CYP2C40, CYP2C8, CYP2S1, CYP51A1, UGT1A3, UGT1A4, UGT1A7, UGT2A1, UGT2B17, UGT2B28, UGT2B7, UGT3A1 |
| Superpathway of Geranylgeranyldiphosphate Biosynthesis | HADHA, ACAT1, ACAT2, FDPS, HADHB, HMGCR, HMGCS2, IDI1, MVD, PMVK |
| Glutathione-mediated Detoxification | GSTT1, GSTA3, GSTA4, GSTA5, GSTM1, GSTM2, GSTM3, GSTM3, GSTM4, GSTM5, GSTT2, GSTT3, MGST1, MGST3 |
| Stearate Biosynthesis I (Animals) | ACOT1, ACOT11, ACOT2, ACOT8, ACSBG1, ACSL1, ACSL3, BDH2, CYP4A11, CYP4A22, DHRS11, ELOVL6, FASN, PORCN, PRXL2B, SLC27A1, SLC27A2 |
| Melatonin Degradation I | UGT1A1, B3GAT3, CYP1A1, CYP1A2, CYP1B1, CYP2A6, CYP2C18, CYP2C40, CYP2C8, CYP2S1, CYP51A1, SULT1D1, UGT1A3, UGT1A4, UGT1A7, UGT2A1, UGT2B17, UGT2B28, UGT2B7, UGT3A1 |
| NRF2-mediated Oxidative Stress Response | GCLC, ABCC2, ACTA1, ACTA2, ACTG2, AOX1, ATF4, ATM, CBR1, CREBBP, DNAJA4, DNAJB9, EIF2AK3, EPHX1, FKBP5, FMO1, FOS, FTH1, FTL, GCLM, GSTA3, GSTA5, GSTM1, GSTM2, GSTM3, GSTM4, GSTM5, GSTM6, GSTT2, HERPUD1, JUN, MAFF, MAP2K6, MGST1, MGST3, NFE2L2, NQO1, NQO2, PIK3CG, PIK3R3, RASD1, RRAS |
| Acetone Degradation I (to Methylglyoxal) | CYP1A1, CYP1A2, CYP1B1, CYP2A6, CYP2C40, CYP2C8, CYP2C18, CYP2S1, CYP4A11, CYP4A22, CYP51A1, DHRS11, PRXL2B |
| GADD45 Signaling | CCNB1, ATM, BRCA1, CCND1, CCND3, CCNE1, CCNE2, CDKN1A, GADD45A |
| **CR-PM vs AL-PM** |  |
| LPS/IL-1 Mediated Inhibition of RXR Function | ABCB1, ABCB11, ABCG5, ABCG8, ACOX1, ACSL1, ACSL3, ALDH1A2, ALDH1B1, ALDH1L2, ALDH3A2, ALDH3B1, CHST3, CPT1A, CPT1B, CPT2, CYP2A6, CYP2B6, CYP2C8, CYP3A5, CYP4A11, CYP4A14 CYP7A1, FABP2, FABP3, FABP4, FABP5, FABP7, FMO2, FMO3, FMO4, FMO5, GSTA3, GSTA5, GSTM1, GSTM2, GSTM3, GSTM4, GSTM5, GSTM6, GSTT2, HMGCS2, HS6ST1, IL18, IL1R1, IL1RN, IL33, IL36G, JUN, LY96, MAOA, MGST3, NGFR, NR0B2, NR1H4, NR1I3, PAPSS2, PLTP, PPARA, PPARGC1A, PPARGC1B, SLC27A1, SLC27A2, SREBF1, SULT1C2, SULT1D1, SULT3A1, SULT3A2, SULT4A1, TNF |
| Superpathway of Cholesterol Biosynthesis | ACAT1, ACAT2, CYP51A1, DHCR7, FDFT1, FDPS, HADHA, HADHB, HMGCR, HMGCS2, HSD17B7, IDI1, LBR, LSS, MVD, NSDHL, PMVK, SC5D |
| PXR/RXR Activation | GSTM1, ABCB1, ABCB11, ALDH3A2, CES3, CPT1A, CYP1A2, CYP2A6, CYP2B6, CYP2C8, CYP3A5, CYP7A1, G6PC, GSTM2, HMGCS2, IGFBP1, NR0B2, NR1I3, PAPSS2, PCK2, PPARA, PPARGC1A, PRKAR1B, PRKAR2B, SCD, TNF, UGT1A7 |
| Calcium Signaling | ACTA2, ACTA1, ASPH, ATP2A1, ATP2B2, CACNA1B, CACNA1S, CACNA2D1, CACNB1, CACNG6, CAMK1, CAMK1D, CAMK2A, CAMK4, CASQ1, CASQ2, CHP1, CHRNA2, CHRNA3, CHRNA4, CHRNE, CREB3L3, GRIN3B, HDAC11, HDAC9, HTR3A, ITPR3, MYH1, MYH2, MYH4, MYH7, MYH7B, MYL1, MYL2, MYL3, MYL4, MYL6B, MYL9, NFATC2, PRKAR1B, PRKAR2B, RCAN1, RCAN2, RYR1, TNNC1, TNNC2, TNNI1, TNNI2, TNNI3, TNNT1, TNNT2, TNNT3, TPM1, TPM2, TRDN, TRPC2 |
| FXR/RXR Activation | SLC22A7, A1BG, ABCB11, ABCB4, ABCG5, ABCG8, ALB, APOA1, APOA4, APOD, C4A/C4B, C9, CYP7A1, CYP8B1, FASN, G6PC, IL18, IL1RN, IL33, IL36G, LPL, MAPK12, NR0B2, NR1H4, PCK2, PKLR, PLTP, PPARA, PPARGC1A, SAA1, SDC1, SERPINA1, SLC10A2, SLC51B, SREBF1, TF, TNF, TTR, VLDLR |
| Fatty Acid β-oxidation I | ACAA1, ACADM, ACSL1, ACSL3, ECI1, ECI2, ECI3, EHHADH, HADHA, HADHB, HSD17B10, HSD17B4, SCP2, SDS, SLC27A1, SLC27A2 |
| GADD45 Signaling | CCNB1, ATM, BRCA1, CCND1, CCNE1, CCNE2, CDK1, CDKN1A, GADD45A, GADD45B, GADD45G, MAP3K4 |
| Aryl Hydrocarbon Receptor Signaling | CYP1B1, ALDH1A2, ALDH1B1, ALDH1L2, ALDH3A2, ALDH3B1, ATM, CCNA2, CCND1, CCNE1, CCNE2, CDKN1A, CDKN2A, CYP1A1, CYP1A2, DHFR, E2F1, FAS, FOS, GSTA3, GSTA5, GSTM1, GSTM2, GSTM3, GSTM4, GSTM5, GSTM6, GSTT2, HSPB2, HSPB7, JUN, MGST3, MYC, NQO1, NQO2, NR0B2, POLA1, TGFB2, TNF |
| Nicotine Degradation II | CYP1A2, B3G AT3, CYP1A1, CYP1B1, CYP2A6, CYP2B6, CYP2C18, CYP2C40, CYP2C8, CYP2E1, CYP3A5, CYP51A1, FMO2, FMO3, FMO4, FMO5, INMT, UGT1A4, UGT1A7, UGT2B17, UGT2B28, UGT2B7, UGT3A1 |
| Xenobiotic Metabolism Signaling | CYP1A1, ABCB1, ALDH1A2, ALDH1B1, ALDH1L2, ALDH3A2, ALDH3B1, ATM, CAMK1, CAMK1D, CAMK2A, CAMK4, CES1G, CES3, CES4A, CHST3, CYP1A2, CYP1B1, CYP2B6, CYP2C8, CYP3A5, EIF2AK3, FMO2, FMO3, FMO4, FMO5, GSTA3, GSTA5, GSTM1, GSTM2, GSTM3,GSTM4, GSTM5, GSTM6, GSTT2, HS6ST1, MAOA, MAP2K6, MAP3K13, MAP3K4, MAP3K6, MAP3K9, MAPK12, MGST3, NQO1, NQO2, NR1I3, PIK3C2G, PPARGC1A, PPP2R2C, PRKCA, RRAS, SULT1C2, SULT1D1, SULT3A1/SULT3A2, SULT4A1, TNF, UGT1A4, UGT1A7, UGT2B17, UGT2B28, UGT2B7 |
| Histidine Degradation VI | CYP11A1, AMDHD1, CYP26A1, CYP26B1, CYP2E1, CYP46A1, CYP4A11, CYP4A22, CYP7B1, HAL |
| AMPK Signaling | SIRT1, ACACA, ACACB, ADIPOQ, ADRA1B, ADRA1D, ADRB1, ADRB2, AK1, AK4, AK6, AK7, ATM, CCNA2, CCND1, CDKN1A, CHRM1, CHRNA2, CHRNA3, CHRNA4, CHRNE, CKM, CPT1A, CPT1B, CPT2, CREB3L3, DPF1, EEF2K, FASN, GYS1, HMGCR, LEP, MAPK12, MLYCD, PCK2, PFKFB2, PFKFB3, PFKFB4, PFKM, PFKP, PIK3C2G, PPARGC1A, PPAT, PPM1D, PPM1K, PPP2R2C, PRKAR1B, PRKAR2B, RAB27A, SLC2A4, SMARCD3 |
| Ketogenesis | ACAT1, ACAT2, BDH2, HADHA, HADHB, HMGCL, HMGCLL1, HMGCS2 |
| Axonal Guidance Signaling | ABLIM3, ACE, ADAM12, ADAM1A, ADAM22, ADAM28, ADAM32, ADAMTS14, ADAMTS15, ADAMTS17, ADAMTS4, ADAMTS7, ADAMTS9, ATM, BDNF, BMP1, BMP10, BMP3, BMP7, CHP1, EFNA3, EFNA5, EPHA1, EPHA2, EPHA3, EPHB6, FES, FZD3, GNA13, GNAI1, GNAO1, GNAT2, ITGA4, KCNJ12, LINGO1, MMP27, MMP28, MMP7, MMP8, MYL1, MYL2, MYL3, MYL4, MYL6B, MYL9, MYLPF, NFATC2, NGEF, NGFR, NRP1, NTF3, NTN3, NTRK2, PAK1, PAK4, PDGFA, PDGFB, PDIA3, PIK3C2G, PLCB4, PLCD4, PLCL1, PLXNA3, PRKAR1B,PRKAR2B,PRKCA,PROK1,ROBO3,RRAS,SEMA3B,SEMA4A,SHANK2, SLIT2, SLIT3, SMO, SOS2, SRGAP2, SRGAP3, TUBA1A, TUBA1B, TUBA1C, TUBA4A, TUBA8, TUBB, TUBB1, TUBB2A, TUBB2B, TUBB3, TUBB4A, TUBB4B, TUBB6, UNC5D, VEGFB, WNT5A, WNT9A |
| LXR/RXR Activation | APOA4, A1BG, ABCG5, ABCG8, ACACA, ALB, APOA1, APOA5, APOD, C4A/C4B, C9, CYP51A1, CYP7A1, FASN, FDFT1, HMGCR, IL18, IL1R1, IL1RN, IL33, IL36G, LDLR, LPL, LY96, NGFR, NR1H4, PLTP, SAA1, SCD, SERPINA1, SREBF1, TF, TNF, TTR, UGT1A3 |
| Estrogen Biosynthesis | CYP2E1, AKR1B7, CYP1A1, CYP1A2, CYP1B1, CYP2A6, CYP2B6, CYP2C18, CYP2C40, CYP2C8, CYP3A5, CYP51A1, HSD17B10, HSD17B11, HSD17B2, HSD17B4, HSD17B7 |
| Mevalonate Pathway I | SULT1D1, ACAT1, ACAT2, HADHA, HADHB, HMGCR, HMGCS2, IDI1, MVD, PMVK |
| Superpathway of Melatonin Degradation | UGT1A4, B3GAT3, CYP1A1, CYP1A2, CYP1B1, CYP2A6, CYP2B6, CYP2C18, CYP2C40, CYP2C8, CYP2E1, CYP3A5, CYP51A1, MAOA, MPO, SULT1D1, UGT1A4, UGT1A7, UGT2B17, UGT2B28, UGT2B7, UGT3A1 |
| Nicotine Degradation III | UGT1A1, B3GAT3, CYP1A1, CYP1A2, CYP1B1, CYP2A6, CYP2B6, CYP2C18, CYP2C40, CYP2C8, CYP2E1, CYP3A5, CYP51A1, UGT1A4, UGT1A7, UGT2B17, UGT2B28, UGT2B7, UGT3A1 |
| Glutathione-mediated Detoxification | GSTT1, GSTA3, GSTA4, GSTA5, GSTM1, GSTM2, GSTM3, GSTM4, GSTM5, GSTT2, GSTT3, MGST3 |

Note: Genes labeled in red were selected for validation of mRNA expression by qPCR (Fig. S15).

**Reference**

1. Myers, J.L. and R.L. Grant, *Development of a chronic inhalation reference value for hexamethylenediamine using an exposure model based on the dihydrochloride salt.* Inhalation Toxicology, 2015. **27**(9): p. 440-449.

2. Li, D., et al., *Multiple organ injury in male C57BL/6J mice exposed to ambient particulate matter in a real-ambient PM exposure system in Shijiazhuang, China.* Environ Pollut, 2019. **248**: p. 874-887.

3. Olive, P.L. and J.P. Banath, *The comet assay: a method to measure DNA damage in individual cells.* Nat Protoc, 2006. **1**(1): p. 23-9.

4. Pugh, T.D., R.G. Klopp, and R. Weindruch, *Controlling caloric consumption: protocols for rodents and rhesus monkeys.* Neurobiology of Aging, 1999. **20**(2): p. 157-165.
